# *In Silico* Integration of Transcriptome and Interactome Predicts an ETP-ALL-Specific Transcriptional Footprint that Decodes its Developmental Propensity

**DOI:** 10.3389/fcell.2022.899752

**Published:** 2022-05-13

**Authors:** Soumyadeep Mukherjee, Arpita Kar, Paramita Paul, Souvik Dey, Avik Biswas, Subhasis Barik

**Affiliations:** ^1^ Department of In Vitro Carcinogenesis and Cellular Chemotherapy, Chittaranjan National Cancer Institute, Kolkata, India; ^2^ Department of Signal Transduction and Biogenic Amines, Chittaranjan National Cancer Institute, Kolkata, India; ^3^ Manipal Centre for Biotherapeutics Research, Manipal Academy of Higher Education, Manipal, India

**Keywords:** ETP-ALL, transcriptome analysis, multipotency, lineage specification, biomarker, personalized medicine, interactome

## Abstract

Early T precursor acute lymphoblastic leukemia (ETP-ALL) exhibits poor clinical outcomes and high relapse rates following conventional chemotherapeutic protocols. Extensive developmental flexibility of the multipotent ETP-ALL blasts with considerable intra-population heterogeneity in terms of immunophenotype and prognostic parameters might be a target for novel therapeutic interventions. Using a public gene expression dataset (GSE28703) from NCBI GEO DataSets with 12 ETP-ALL and 40 non-ETP-ALL samples, such heterogeneity was found to be reflected in their transcriptome as well. Hub genes were identified from the STRING-derived functional interaction network of genes showing differential expression between ETP-ALL and non-ETP-ALL as well as variable expression across ETP-ALL. Nine genes (*KIT*, *HGF*, *NT5E*, *PROM1*, *CD33*, *ANPEP*, *CDH2*, *IL1B,* and *CXCL2*) among the hubs were further validated as possible diagnostic ETP-ALL markers using another gene expression dataset (GSE78132) with 17 ETP-ALL and 27 non-ETP-ALL samples. Linear dimensionality reduction analysis with the expression levels of the hub genes in ETP-ALL revealed their divergent inclinations towards different hematopoietic lineages, proposing them as novel indicators of lineage specification in the incompletely differentiated ETP-ALL blasts. This further led to the formulation of a personalized lineage score calculation algorithm, which uncovered a considerable B-lineage-bias in a substantial fraction of ETP-ALL subjects from the GSE28703 and GSE78132 cohorts. In addition, STRING-derived physical interactome of the potential biomarkers displayed complete segregation of the B-lineage-skewed markers from other lineage-associated factors, highlighting their distinct functionality and possible druggability in ETP-ALL. A panel of these biomarkers might be useful in pinpointing the dominant lineage specification programmes in the ETP-ALL blasts on a personalized level, urging the development of novel lineage-directed precision therapies as well as repurposing of existing therapies against leukemia of different hematopoietic lineages; which might overcome the drawbacks of conventional chemotherapy.

## Introduction

T cell acute lymphoblastic leukemia (T-ALL) is an aggressive hematologic neoplasm of the T lymphocytic compartment, accounting for 15 and 25% of total pediatric and adult acute lymphoblastic leukemia (ALL) cases, respectively (Vadillo et al., 2018). The disease mostly involves cancerous transformation of the T-lineage primed progenitors (Kraszewska et al., 2012), reflected in its immunophenotypic resemblance to that of the T cells (Bene et al., 1995). Interestingly, early T precursor acute lymphoblastic leukemia (ETP-ALL), a recently discovered subgroup of T-ALL, shows a considerably divergent immunophenotype, including low levels of T-lineage commitment markers along with high expression of one or more stem cell or myeloid antigens (Coustan-Smith et al., 2009; Chopra et al., 2014). The leukemic blasts of ETP-ALL arise from a bone marrow-derived multipotent hematopoietic progenitor called early thymic progenitor (ETP), designated as the earliest precursor of thymic T-lymphocytes (Treanor et al., 2014; Booth et al., 2018). In spite of persisting debates regarding the deterministic ontogeny of the ETPs, their capability of producing cells of myeloid (Bell and Bhandoola, 2008; Wada et al., 2008; Luc et al., 2012) as well as B-lymphocytic (Luc et al., 2012) lineages have been undisputed. A spectrum of potencies exists within the ETP population, often marked by lineage-specific signatures (Haymaker et al., 2012), which gives rise to considerable intra-population heterogeneity among the ETPs and influences their developmental outcome according to microenvironmental cues (Barik et al., 2017; Barik et al., 2018). This multitude of potencies is reflected in the variability of lineage-restricted marker expression in the ETP-ALL blasts on a patient-to-patient basis.

In spite of a relatively low rate of incidence among children (11%) as well as adults (7.4%), the clinical outcome for ETP-ALL is often remarkably poor; even worse than other T-ALL subtypes (Coustan-Smith et al., 2009; Inukai et al., 2012). However, chemotherapy is the only available tool in defence of this disease due to the paucity of United States Food and Drug Administration (FDA)-approved targeted therapies (Castaneda Puglianini and Papadantonakis, 2020). Conventional chemotherapeutic strategies to combat T-ALL exhibit suboptimal clinical efficiency against ETP-ALL (Wood et al., 2014; Jain et al., 2016; Yang et al., 2019). The presence of minimal residual disease and the resultant chances of relapse after such chemotherapeutic interventions constitute a point of serious concern regarding this disease (Wood et al., 2009; Zhang et al., 2020). Therefore, making a successful diagnosis is crucial for the clinical efficiency in case of ETP-ALL treatment, since being misdiagnosed as a case of generic T-ALL might lead to application of fallacious therapies.

Several studies have explored the genetic traits of ALL samples and have identified various mutational and transcriptional signatures unique to ETP-ALL (Zhang et al., 2012; Neumann et al., 2013; Zuurbier et al., 2014; Kumar et al., 2019; Wang and Zhang, 2020). Although these biomarkers successfully discriminate ETP-ALL from other incidences of T-ALL, one important caveat still remains: the developmental plasticity of the ETP-ALL blasts. Several targeted therapies against ETP-ALL are currently under evaluation, such as Peg-L-Asparaginase (Patrick et al., 2014), Ruxolitinib (Maude et al., 2015; Verbeke et al., 2019), Venetoclax (Numan et al., 2018), Venetoclax-plus-Nelarabine (McEwan et al., 2020) etc., with demonstrated efficacies against the disease. However, because of the miscellany of potencies in possession of the ETP-ALL blasts, any single therapeutic regimen being universally effective against every single case of ETP-ALL is extremely unlikely, if at all possible. As a result, an individual with majority of B-lineage-biased ETP-ALL blasts may not show satisfactory remission upon treatment with myeloid-targeted therapies, owing to the subtle differences in a vast range of physiological responses of cells from different hematopoietic lineages against the same drug. In fact, different cases of ETP-ALL show different responses against similar kind of therapies (Bernt et al., 2016; Genescà et al., 2020; McEwan et al., 2020; Sin and Man, 2021). Again, the lack of clarity regarding the expression of lineage markers in ETP-ALL often leads to misclassification of individual ETP-ALL cases with aberrant non-T marker expression as non-T-lineage neoplasms (Khurana et al., 2020). Therefore, the so far underappreciated multi-lineage potency of the ETP-ALL blasts might be targeted to aid the diagnosis of as well as the therapy designing against this malignant pathology, especially as a stepping stone for precision medicine (Tarantini et al., 2021).

Against such a backdrop, this study explored the ETP-ALL transcriptome available on public databases to pinpoint the hubs from a functional interaction network of genes showing significant difference in expression between ETP-ALL and non-ETP-ALL subjects as well as substantial variance among the ETP-ALL subjects themselves. Transcript levels of the genes, therefore, might not only classify ALL into ETP-ALL and non-ETP-ALL, but might further categorize ETP-ALL into separate subclasses based on their differential lineage inclinations. This may potentiate the invention of novel precision therapies targeting different lineage propensities, which might eliminate the shortcomings of conventional chemotherapy. Besides, the findings might open new avenues for the prospective use of B-cell acute lymphoblastic leukemia (B-ALL) or acute myelogenous leukemia (AML)-directed therapeutic modalities against ETP-ALL in a case-by-case manner.

## Materials and Methods

### Data Acquisition and Processing

**FIGURE 1:**
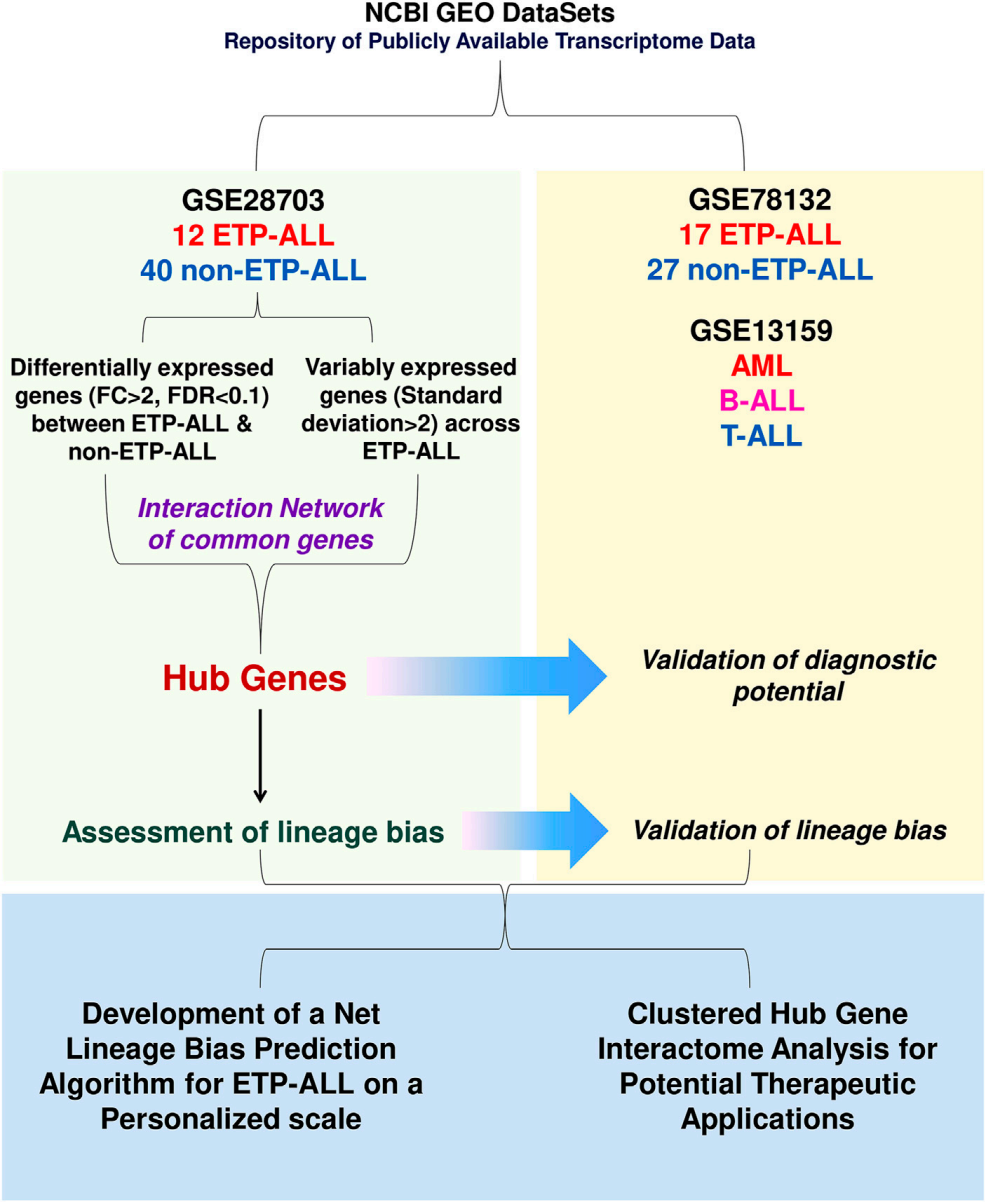
Diagrammatic representation of the work-flow. The entire work lies on three segments: discovery of transcriptomic signatures from the GSE28703 dataset, validation of the proposed signatures in the GSE78132 (and GSE13159 in some cases) datasets and amalgamation of the validated findings in order to identify potential lineage-directed diagnostic and therapeutic implementations against ETP-ALL.

The flow of work adopted in this study is depicted in Figure 1. Two publicly available transcriptome datasets (accession numbers: GSE28703 and GSE78132) containing normalized gene expression data from ETP-ALL and non-ETP-ALL leukemic blasts were selected from NCBI Gene Expression Omnibus (GEO) DataSets (Barrett et al., 2012) after a search against the keyword “ETP-ALL”. The GSE28703 data, comprising of 12 ETP-ALL and 40 non-ETP-ALL samples, was used to discover differential expression signatures between ETP-ALL and non-ETP-ALL; while the GSE78132 data (GPL96 platform), comprising of 17 ETP-ALL and 27 non-ETP-ALL samples, was used for validation of the identified signatures. The relatively smaller size of the patient cohorts was accepted due to the relatively rare occurrence rate of ETP-ALL (Coustan-Smith et al., 2009). Another public microarray dataset (accession number: GSE13159) was used to evaluate gene expression levels in acute leukemia of mature leukocytes (T-ALL, B-ALL, AML) as well as progenitor/precursor-B-cell acute lymphoblastic leukemia (pro/pre-B-ALL). Detailed information regarding the datasets is provided in Supplementary Table S1. Probe annotations and further processing of the datasets were performed as previously described (Mukherjee et al., 2021).

### Clustering

The unsupervised, agglomerative hierarchical clustering-based dendrogram, constructed on iDEP.92 (Ge et al., 2018) with top 10,000 variable genes, clustered samples on the basis of Euclidean distance and complete linkage. Multidimensional scaling (MDS)-based clustering was conducted on iDEP.92 as a method of non-linear dimensionality reduction.

### Differential Gene Expression Analysis

Identification of differentially expressed genes (DEG) between sample groups was performed on iDEP.92 using the limma function with fold-change >2 and FDR (false discovery rate) <0.1. Genes with standard deviation >2 in expression values within the ETP-ALL group were selected as variably expressed genes within ETP-ALL. Gene expression heatmaps of differentially/variably expressed genes were created on Heatmapper (Babicki et al., 2016). Venn diagram of differentially regulated genes was built using InteractiVenn (Heberle et al., 2015).

### Protein-Protein Interaction Network Construction

Knowledge-based protein-protein interaction maps involving genes of interest were retrieved from STRING (Szklarczyk et al., 2019) and visualized on Cytoscape v3.8.2 (Shannon et al., 2003). Each node represented a protein, while each edge represented an interaction between them. For functional networks, these edges included every possible interaction criterion (gene neighborhood, gene fusion, gene co-occurrence, co-expression etc), whereas for physical networks, the edges signified physical interactions only. For all networks, a confidence cut-off of 0.4 was taken for edge mapping. Only the nodes connected to at least one edge were visualized on the network. Hub genes were identified using the cytoHubba plugin (Chin et al., 2014). Maximal clique centrality (MCC) scores were considered the key metric while ranking the genes in terms of their quality to act as hub genes within the network.

### Enrichment Analysis

Over-representation analysis with the differentially expressed genes between ETP-ALL and non-ETP-ALL was performed on ConsensusPathDB (Kamburov et al., 2009; Kamburov et al., 2011) against the ‘REACTOME’ database (for significantly enriched pathways) and on Enrichr (Chen et al., 2013; Kuleshov et al., 2016; Xie et al., 2021) against the ‘CORUM’ database (for significantly enriched protein complexes). Pathways/protein complexes with *p*-value <0.01 and overlap >5 genes were mapped onto the pathway network. Edge thicknesses on the networks described the relative overlap in genes between the two gene set nodes. Gene set enrichment analysis (GSEA) against the ‘GO biological process’ (for pathways) and ‘TF.Target.TRRUST’ (for transcription factors) databases for ETP-ALL samples with higher and lower levels of hub gene expression than the corresponding cohort mean was performed on iDEP.92, with FDR <0.2. Pathway enrichment on Cytoscape was carried out using the ‘STRING Enrichment’ function on the ‘StringApp’ plugin (Doncheva et al., 2018). The whole genome network was used as the background, while the ‘STRING Clusters’ database was used as the reference for pathway mappings in the converged network. ‘REACTOME pathways’ database was used for pathway mappings in the networks of individual hub genes. The ‘MSigDB hallmark’ and ‘CORUM’ databases on Enrichr were used as reference for pathway enrichment and protein complex enrichment, respectively, of genes variably expressed among ETP-ALL samples. All enrichment analyses were carried out assuming hyper-geometric probabilistic distributions of the reference gene sets within the respective gene lists in each case.

### Classifier Performance Analysis

The ability of genes as binary classifiers in discriminating between ETP-ALL and non-ETP-ALL was tested using receiver operating characteristic (ROC) analysis on ROCplot (Fekete and Győrffy, 2019). Magnitude of the area under curve (AUC) statistic was considered representative of classifier quality. Corresponding confusion matrices denoted the extent of true and false prediction rates in the classification process for individual classifiers.

### Association Analysis

The pairwise correlation matrix of all ETP-ALL and non-ETP-ALL samples, constructed on iDEP.92, was composed of Pearson’s correlation coefficients between each pair of samples, denoting their whole transcriptomic correlation. Linear dimensionality reduction in terms of principal component analysis (PCA) with normalized expression values of selected genes in ETP-ALL was carried out on ClustVis (Metsalu and Vilo, 2015). Normalization of expression values of individual genes was performed with respect to their cognate mean expression levels in the non-ETP-ALL group to yield a measure of co-expression trends across different lineages. For analyzing associations of individual hub gene expression with particular lineages, the ETP-ALL cohort was divided into two groups based on higher and lower expression levels of the individual hub genes compared to their corresponding mean expression, followed by analysis of lineage marker expression in these groups. Correlation between the expression levels of individual genes and the leukemia subtypes based on GSE13159 data was observed on BloodSpot (Bagger et al., 2019).

### Personalized Lineage Score Formulation

For prediction of personalized scores indicating net lineage skew in leukemic blasts from ETP-ALL patients, expression values of each hub gene were normalized with respect to the population mean expression values of the respective genes. Next, the normalized expression values were transformed to a fold-change value with respect to the stem-like condition by the following formula:

Transformed Expression Score=Normalized expression score of hub gene÷Normalized expression score of PROM1

Lineage scores were further computed as geometric means of transformed expression scores of all genes displaying skew towards individual lineages (adapted from Ravichandran et al., 2021). Formulae for calculation of the respective lineage scores are as follows (TES: Transformed Expression Score).

$$Myeloid\;lineage\;score={\left[TES\left(KIT\right)\times TES\left(HGF\right)\times TES\left(ANPEP\right)\times TES\left(CXCL2\right)\right]}^{1/4}$$

$$B-lineage\;score={\left[TES\left(NT5E\right)\times TES\left(CXCL2\right)\right]}^{1/2}$$

T−lineage score=TES(CDH2)

$$Lineage\;score\;for\;unidentified\;bias={\left[TES\left(CD33\right)\times TES\left(IL1B\right)\right]}^{1/2}$$

All lineages with lineage score values within 10% error range of the maximum lineage score for an individual sample were assigned to the respective sample.

### Statistics and Data Visualization

Statistical analyses were carried out on GraphPad Prism v5.0 for Windows, GraphPad Software, La Jolla, California, United States. Column plots were constructed using the mean and standard error of mean (SEM). Box plots represented the mean and interquartile range. Whiskers extended till the 5th and 95th percentile, while remaining points were shown as outliers. Violin plots, constructed on BoxPlotR (Spitzer et al., 2014), additionally represented the Kernel density distributions aligned sidewise. For statistical comparisons, two-tailed unpaired Student’s t-test with 95% confidence interval was performed. Benjamini–Hochberg procedure was applied to compute false discovery rates for multiple comparisons (Benjamini and Hochberg, 1995). Bubble plots represented the overlap between test and reference gene sets and -log_10_FDR along the axes, while bubble diameters were proportional to gene set sizes.

## Results

### ETP-ALL Blasts Exhibit Pronounced Transcriptomic Differences in Comparison With Non-ETP-ALL

**FIGURE 2:**
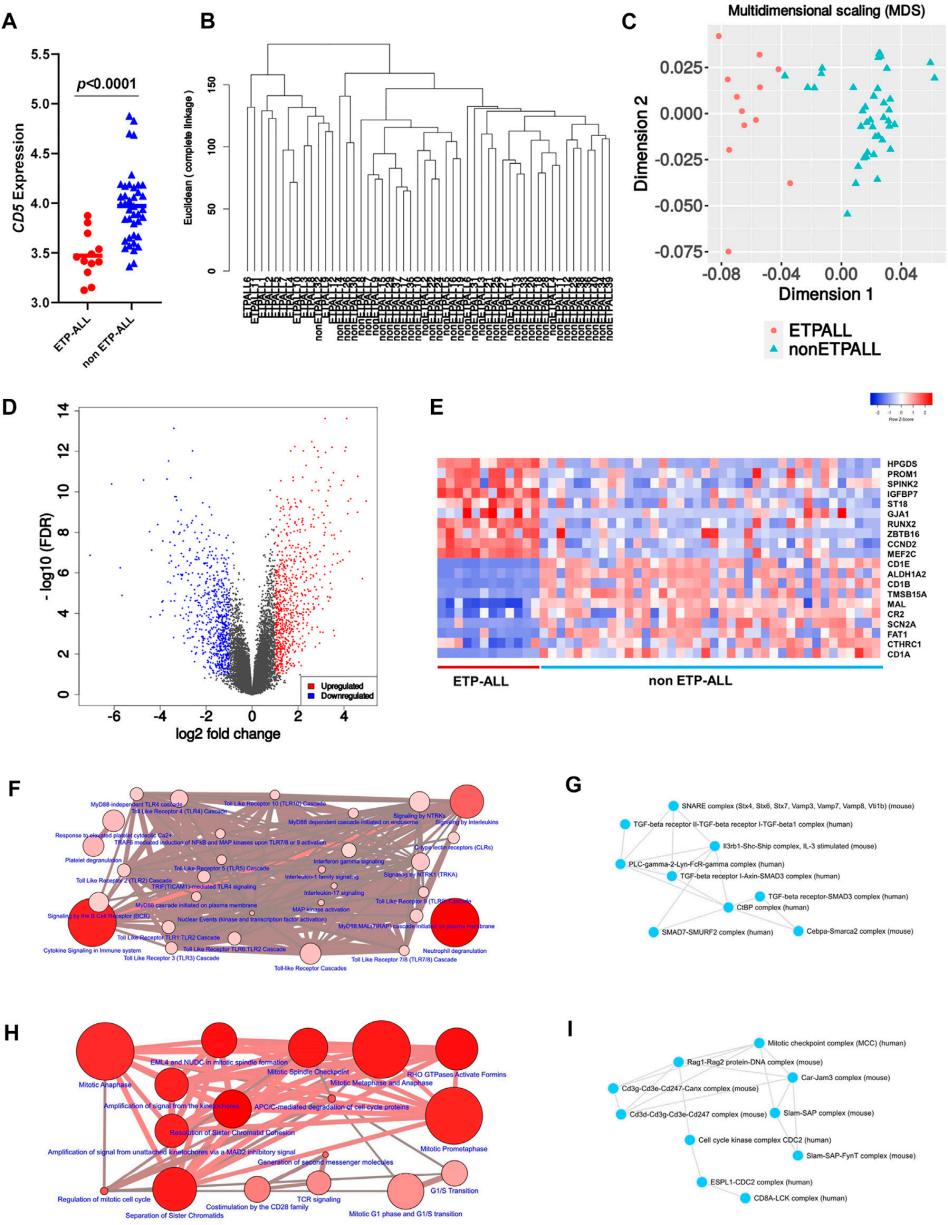
Contrasting transcriptomic features between ETP-ALL and non-ETP-ALL. **(A)** Expression level of CD5 in ETP-ALL and non-ETP-ALL groups from GSE28703 dataset. Statistical significance was calculated using two-tailed unpaired Student’s t-test. **(B)** Rooted dendrogram of all samples from GSE28703 dataset showing unsupervised hierarchical clustering between ETP-ALL and non-ETP-ALL. **(C)** Multidimensional scaling (MDS) plot of ETP-ALL (red circle) and non-ETP-ALL (cyan triangle) samples from GSE28703. **(D)** Volcano plot representing differentially expressed genes (fold change >2, false discovery rate <0.1) between ETP-ALL and non-ETP-ALL groups from GSE28703. Red dots represent genes upregulated, while blue dots represent genes downregulated in ETP-ALL compared to non-ETP-ALL. **(E)** Row z-score normalized unclustered heatmap of expression levels of top 20 differentially expressed genes between ETP-ALL and non-ETP-ALL groups from GSE28703. **(F)** Network of REACTOME pathways enriched in genes upregulated in ETP-ALL. Diameter and colour intensity of nodes represent gene set size and statistical confidence, respectively. **(G)** Network of protein complexes from CORUM enriched in genes upregulated in ETP-ALL. **(H)** Network of REACTOME pathways enriched in genes downregulated in ETP-ALL (upregulated in non-ETP-ALL). **(I)** Network of protein complexes from CORUM enriched in genes downregulated in ETP-ALL.

To design transcriptome-based diagnostic signatures of ETP-ALL, the first step was to test if the immunophenotypic differences between ETP-ALL and non-ETP-ALL (Coustan-Smith et al., 2009) are reflected in their transcriptome. Quality of the processed data from the discovery dataset (GSE28703) was assessed by checking the box plot (Supplementary Figure S1A) and density distribution (Supplementary Figure S1B) of normalized expression values from individual samples. In addition, significantly different *CD5* expression between the two groups (Figure 2A) supported their respective ETP-ALL and non-ETP-ALL status (Chopra et al., 2014). Unsupervised hierarchical clustering on a rooted dendrogram (Figure 2B) showed clear divergence between the two groups. Only one non-ETP-ALL sample was present as an outlier in the ETP-ALL branch, while the non-ETP-ALL branch carried no contamination from the ETP-ALL group. Similarly, discernible clustering between the two ALL groups was observed on the multidimensional scaling plot (Figure 2C). Such distinguishable transcriptomic patterns between ETP-ALL and non-ETP-ALL not only supported the biological differences between them, but also pointed out the necessity to acknowledge the biological properties of ETP-ALL as a separate entity in order to design prospective therapies against the disease. The extent of upregulated and downregulated transcripts (Figure 2D) was comparable between the two groups, indicating no global transcriptional shift in any of them. This idea of balanced transcriptomic alterations was further supported by the MA plot (Supplementary Figure S1C) and scatter-plot of average expression values in the two ALL groups (Supplementary Figure S1D), showing near-similar counts (Supplementary Figure S1E) of DEGs (Supplementary Table S2) in both directions. Genes showing higher expression in ETP-ALL were mostly related to non-T lineages as well as hematopoietic stemness, while many of the genes showing elevated expression in non-ETP-ALL were associated with T-lineage commitment (Figure 2E). These transcriptomic contrasts underpinned the pathway-level disparity between the two groups. ETP-ALL blasts showed enrichment in Toll like receptor (TLR) cascades and pro-inflammatory cytokine (Interleukin-1, Interleukin-17, Interferon-γ) signalling pathways (Figure 2F, Supplementary Table S3), likely to be mediated by inflammatory protein complexes containing Transforming Growth Factor (TGF)-β, Phospholipase C-γ etc (Figure 2G, Supplementary Table S4). Non-ETP-ALL blasts displayed higher propensity towards cell cycle progression, cell division as well as T-cell receptor-mediated and co-stimulatory signalling events (Figure 2H, Supplementary Table S3), where cell cycle checkpoint complexes and T cell maturation factors such as CD3, RAG (Recombination activating gene) clusters plausibly play a pivotal part (Figure 2I, Supplementary Table S4).

### ETP-ALL Bears Considerable Intra-Group Transcriptomic Diversity

**FIGURE 3:**
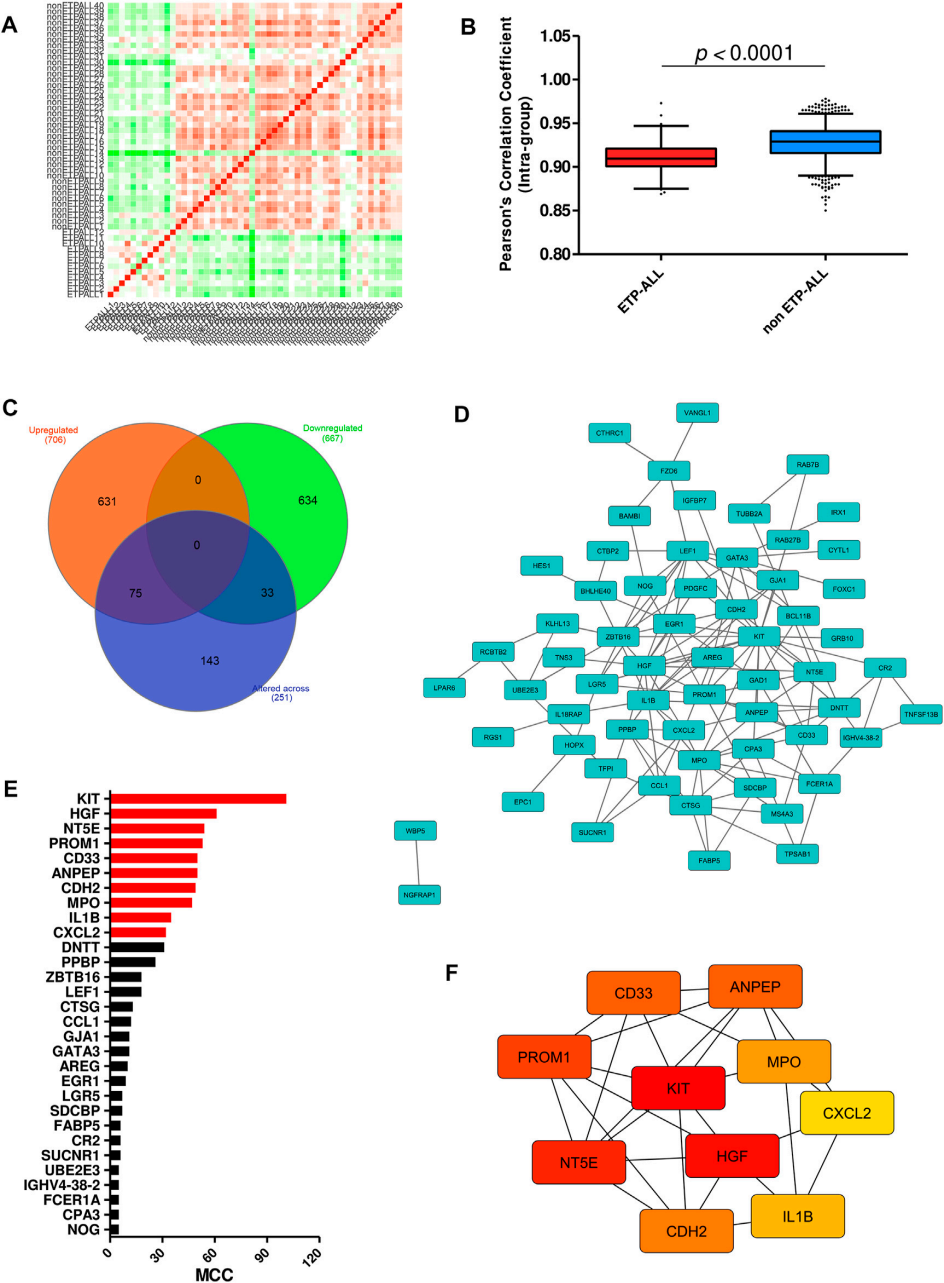
Transcriptomic variability within the ETP-ALL group. **(A)** Pair-wise correlation matrix of global gene expression levels between each sample from GSE28703. **(B)** Box-plot of intra-group Pearson’s correlation coefficients in ETP-ALL and non-ETP-ALL groups. Statistical significance was calculated using two-tailed unpaired Student’s t-test. **(C)** Venn diagram of different sets of differentially expressed genes. Tangerine, green and purple circles represent genes upregulated in, downregulated in and altered (standard deviation >2) across ETP-ALL, respectively. **(D)** Functional protein-protein interaction network of genes up/downregulated in as well as altered across ETP-ALL. Constructed on Cytoscape v3.8.2. **(E)** Maximal Clique Centrality (MCC) score of top 30 hub nodes from the network in Figure 3D. Top 10 hub nodes are labelled in red. **(F)** Shortest interaction path of top 10 hub genes within the network. Constructed on Cytoscape v3.8.2.

Interestingly, a high fraction of subjects among the ETP-ALL group showed lower transcriptome-wide correlation (Figure 3A), while transcriptome from most of the non-ETP-ALL cases were tightly correlated among themselves; yielding a statistically significant difference between the distributions of their correlation coefficients (Figure 3B). Many of the top genes showing very high standard deviations among the ETP-ALL cases were associated with various hematopoietic lineages (Supplementary Figure S2A). Top pathways (Supplementary Figure S2B, Supplementary Table S5) and protein complexes (Supplementary Figure S2C, Supplementary Table S6) associated with these genes, expected to show variable activities across the ETP-ALL cohort, were of diverse functional categories; starting from cell survival and apoptosis to metabolism and hemostasis. Among the 251 genes (Supplementary Table S7) contributing most to this intra-group variability of ETP-ALL, 75 and 33 genes, respectively, exhibited higher and lower transcript levels in ETP-ALL compared to non-ETP-ALL (Figure 3C, Supplementary Table S8). These 108 (75 + 33) genes, supposedly at the root of the transcriptomic heterogeneity within the ETP-ALL group, were also differentially expressed between ETP-ALL and non-ETP-ALL, ensuring this variability to be ETP-ALL-specific. To identify potential key drivers effectuating this transcriptomic diversity of ETP-ALL, the 108 genes were integrated into a functional protein-protein interaction (PPI) network (Figure 3D). Based on maximal clique centrality (MCC) scoring (Supplementary Table S9), top 10 nodes were selected as hub genes (Figure 3E), which displayed the highest degree of centrality across the entire network, therefore could be targeted to achieve maximal success in perturbing the network. An appreciable level of connectivity among the hub genes themselves (Figure 3F) supported their operational centrality.

### Most of the Hub Genes Exhibit Contrasting Expression Between ETP-ALL and Non-ETP-ALL

**FIGURE 4:**
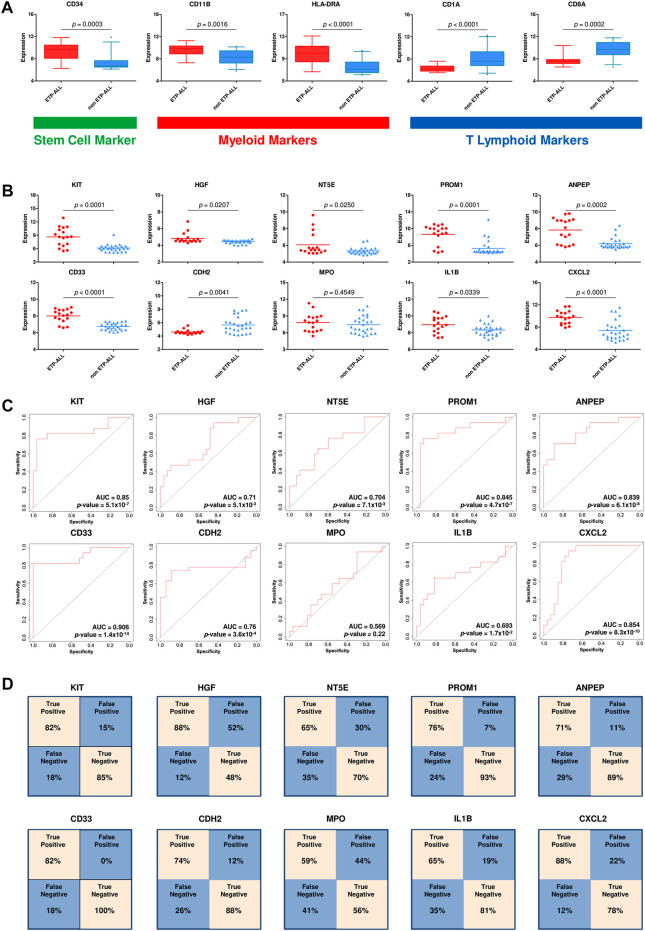
Validation of the hub genes as diagnostic biomarkers of ETP-ALL. **(A)** Expression levels of conventional ETP-ALL markers in ETP-ALL and non-ETP-ALL groups from GSE78132 dataset. Statistical significance was calculated using two-tailed unpaired Student’s t-test. **(B)** Expression levels of hub genes in ETP-ALL and non-ETP-ALL groups from GSE78132 dataset. Two-tailed unpaired Student’s t-test was used to estimate the statistical significance. **(C)** Receiver operating characteristic (ROC) curves of hub genes. AUC (area under curve) values indicate prediction accuracy of the genes as biomarkers. **(D)** Confusion matrices denoting classification qualities of hub genes. For all hub genes apart from CDH2, ‘True positive’ indicates % of ETP-ALL cases with higher expression than ROC cutoff; ‘False negative’ indicates % of ETP-ALL cases with lower expression than ROC cutoff; ‘False positive’ indicates % of non-ETP-ALL cases with higher expression than ROC cutoff; ‘True negative’ indicates % of non-ETP-ALL cases with lower expression than ROC cutoff. The annotations are opposite for CDH2.

After requisite quality assessments (Supplementary Figure S3), a second dataset (GSE78132) was analyzed to affirm the distinctive expression patterns observed in the discovery dataset. Compared to non-ETP-ALL samples, the ETP-ALL group expressed higher levels of stem cell marker *CD34* as well as myeloid markers *CD11B* and *HLA-DRA*, and lower levels of T cell commitment markers *CD1A* and *CD8A*, in this dataset (Figure 4A), concordant with the expected trends. Nine of the ten hub genes exhibited statistically significant differences in expression between the two groups, showing good agreement with the findings from the discovery dataset (Figure 4B). AUC parameters for six of the nine hub genes (*KIT*, *PROM1*, *ANPEP*, *CD33*, *CDH2* and *CXCL2*) on ROC plots were comprehensively higher (>0.75) than those of the others (Figure 4C). Three genes among the latter four, apart from *MPO*, showed satisfactory AUC values (>0.65). Reliability of the classification performances by the genes between ETP-ALL and non-ETP-ALL in terms of true positivity rate and true negativity rate, as observed on the corresponding confusion matrices (Figure 4D), was also in the range of reasonable to very good. Therefore, these nine genes (*KIT*, *PROM1*, *ANPEP*, *CD33*, *CDH2*, *CXCL2, HGF, NT5E,* and *IL1B*) were selected as candidate diagnostic biomarkers of ETP-ALL.

### Expression Levels of the Proposed ETP-ALL Biomarkers are Indicative of Their Developmental Bias

**FIGURE 5:**
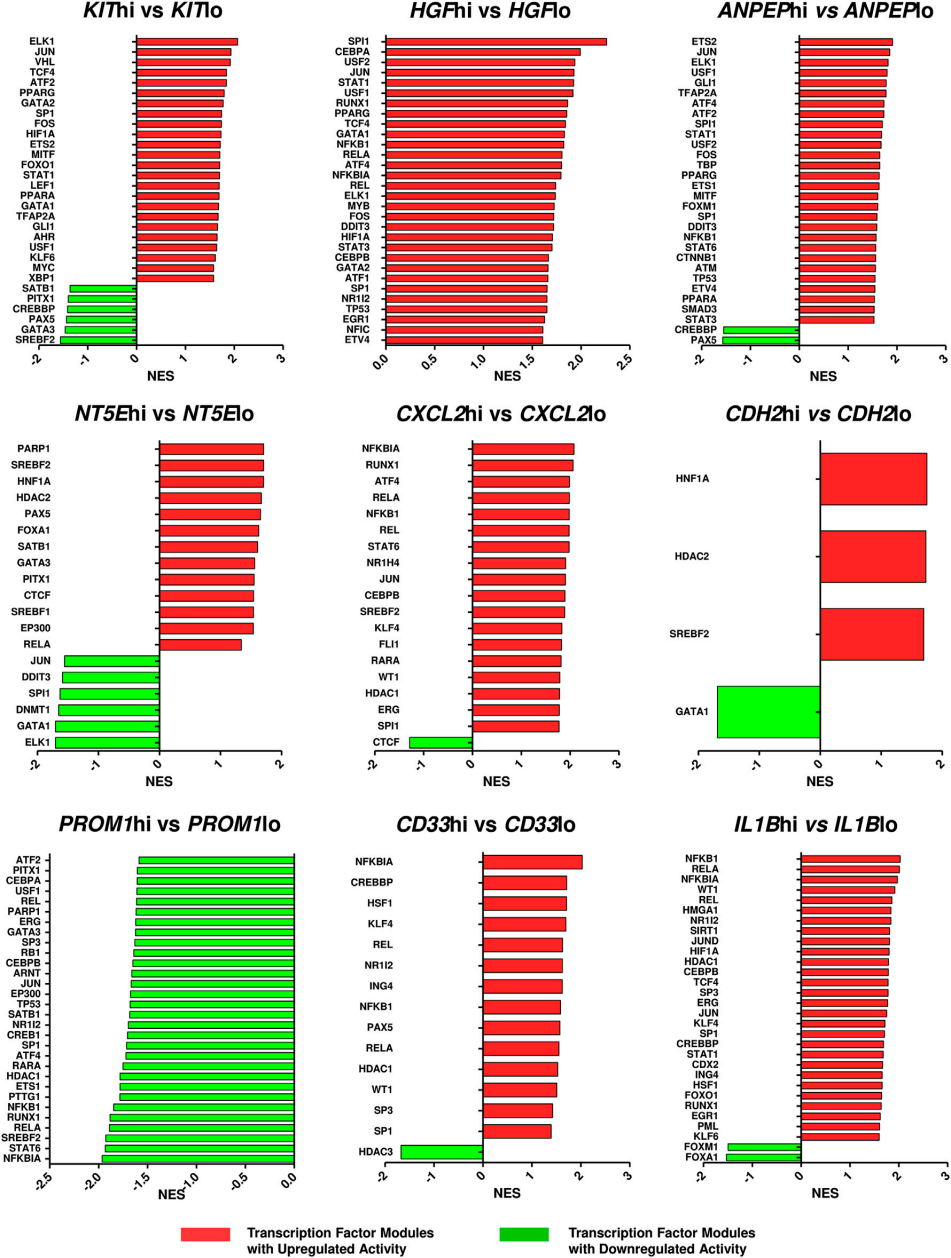
Altered activities of transcription factors across ETP-ALL subpopulations expressing different levels of the hub genes. The ETP-ALL cohort from the GSE28703 dataset was divided into subpopulations on the basis of high and low levels of individual hub gene expression (details in ‘Enrichment analysis’ under ‘Materials & Methods’), followed by GSEA against the ‘TF.Target.TRRUST’ database. Normalized enrichment score (NES) of top 30 transcription factor modules, at most, were represented on the column plots.

**FIGURE 6:**
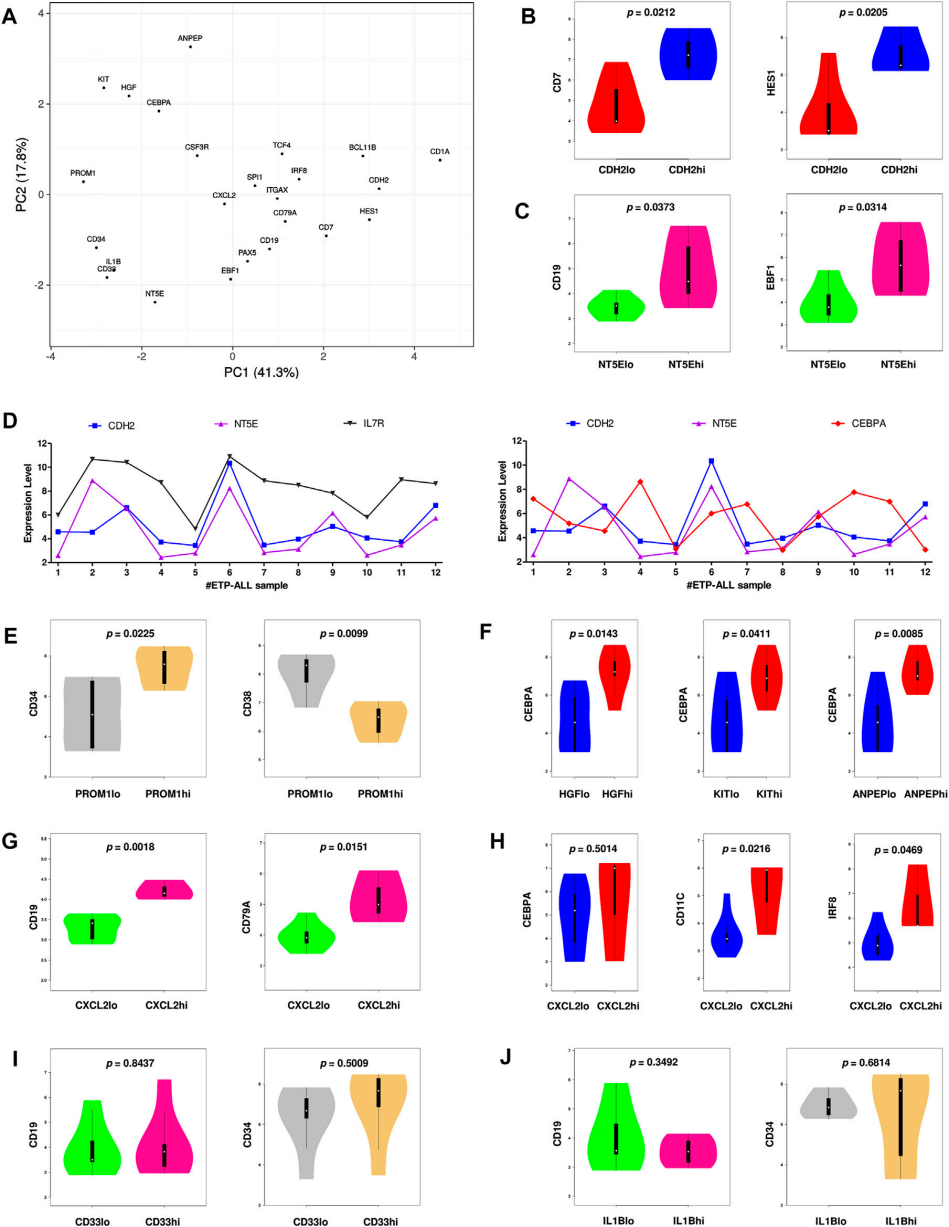
Inclination of the proposed biomarkers towards different hematopoietic lineages. **(A)** Principal component analysis (PCA) plot of normalized expression scores (from GSE28703) of the hub genes along with standard markers of hematopoietic lineages. Lineage affiliation of the known markers are as follows; CD34: stemness; CEBPA, CSF3R: myelo-monocytic; EBF1, PAX5, CD19, CD79A: B-lineage; CD7, HES1, BCL11B, CD1A: T-lineage; SPI1, ITGAX, IRF8, TCF4: dendritic cells. **(B)** Expression of CD7 and HES1 along with different levels of CDH2. **(C)** Expression of CD19 and EBF1 along with different levels of NT5E. **(D)** Expression of CDH2, NT5E, IL7R, and CEBPA across different ETP-ALL samples from GSE28703. **(E)** Expression of CD34 and CD38 along with different levels of PROM1. **(F)** Expression of CEBPA along with different levels of HGF, KIT and ANPEP. **(G)** Expression of CD19 and CD79A along with different levels of CXCL2. **(H)** Expression of CEBPA, CD11C, and IRF8 along with different levels of CXCL2. **(I)** Expression of CD19 and CD34 along with different levels of CD33. **(J)** Expression of CD19 and CD34 along with different levels of IL1B. Two-tailed unpaired Student’s t-test was used to compute the statistical significance for each comparison.

Since the hub genes were pivots of the transcriptomic variations across ETP-ALL samples, it was hypothesized that these genes might have crucial involvements in the multi-lineage diversification of the ETP-ALL blasts. Bisection of the GSE28703 ETP-ALL cohort based on individual hub gene expression yielded many genes with differential expression across subpopulations (Supplementary Figure S4). Subsequent gene set enrichment analyses between ETP-ALL subpopulations with high and low expression levels of each hub gene revealed marked alterations in several pathways (Supplementary Figure S5) and transcription factor activities (Figure 5) of hemato-developmental significance. Such observations hinted towards the fact that variable expression of these genes might act as signatures of different hematopoietic lineage induction processes in the multipotent ETP-ALL blasts. To further establish the relationship between hub gene expression and lineage fate decisions, the hub genes as well as certain developmental markers (transcription factors as well as surface proteins) relevant to different hematopoietic branches were overlaid on the two-dimensional PCA plane (Figure 6A), where proximity between genes was proportional to the extent of their co-expression within the ETP-ALL cohort from the GSE28703 dataset. The assumed associations between the hub genes and the respective lineages were validated by the expression levels of lineage marker genes in the bisected subpopulations within the ETP-ALL cohort. Expression of T-lineage markers *CD7* and *HES1* varied in concordance with *CDH2* expression (Figure 6B), while B-lineage markers *CD19* and *EBF1* significantly varied with *NT5E* levels (Figure 6C). Signature of lymphoid commitment at the expense of myeloid potency was ascertained by the expression trends of *IL7R* (common lymphoid marker) and *CEBPA* (myeloid marker) along with *CDH2* and *NT5E* in individual ETP-ALL subjects (Figure 6D). Despite their common lymphoid bias, *CDH2* and *NT5E* did not affect B- and T-lineage marker expression, respectively (Supplementary Figures S6A,B), while showing a common reciprocal trend with myeloid-associated gene expression (Supplementary Figure S6C). *NT5E,* additionally, showed a quasi-significant (0.05 < *p* < 0.1) inverse correlation with stemness marker *CD34* (Supplementary Figure S6D), its close neighbour on the PCA plot. *PROM1* expression exhibited positive correlation with its nearest neighbour *CD34*, along with a decline in the levels of *CD38* (Figure 6E) as well as the lineage markers (Supplementary Figure S6E) in the *PROM1*hi subgroup. The myelo-monocytic marker *CEBPA*, appearing in the vicinity of *HGF*, *KIT* and *ANPEP*, displayed robust association with the expression levels of these genes (Figure 6F). Concomitantly, the lymphoid markers exhibited an inverse trend with respect to these three hub genes (Supplementary Figures S6F,G). *CXCL2*, juxtaposed to B- as well as myeloid-lineage markers on the PCA plot, showed significant agreement with the levels of B-lineage-specific (Figure 6G) as well as myeloid-oriented (Figure 6H) markers. However, the myeloid predisposition of *CXCL2* was more pronounced for dendritic cell-specific markers *CD11C* and *IRF8* than granulo-monocytic marker *CEBPA*. Neither *CD33* (Figure 6I) nor *IL1B* (Figure 6J) exhibited consistency with the expression levels of their nearest neighbour *CD34* as well as *CD19*, which, despite being distant on the PCA plane, was assumed to have a probable association with these genes due to their proximity to *NT5E*. Replication of these observations in the validation cohort (GSE78132) reinforced the proposed biomarkers as indices of lineage bias in ETP-ALL (Supplementary Figure S7).

### Developmental Skew of the Proposed ETP-ALL Biomarkers are Corroborated by Their Expression Levels in B-ALL and AML

**FIGURE 7:**
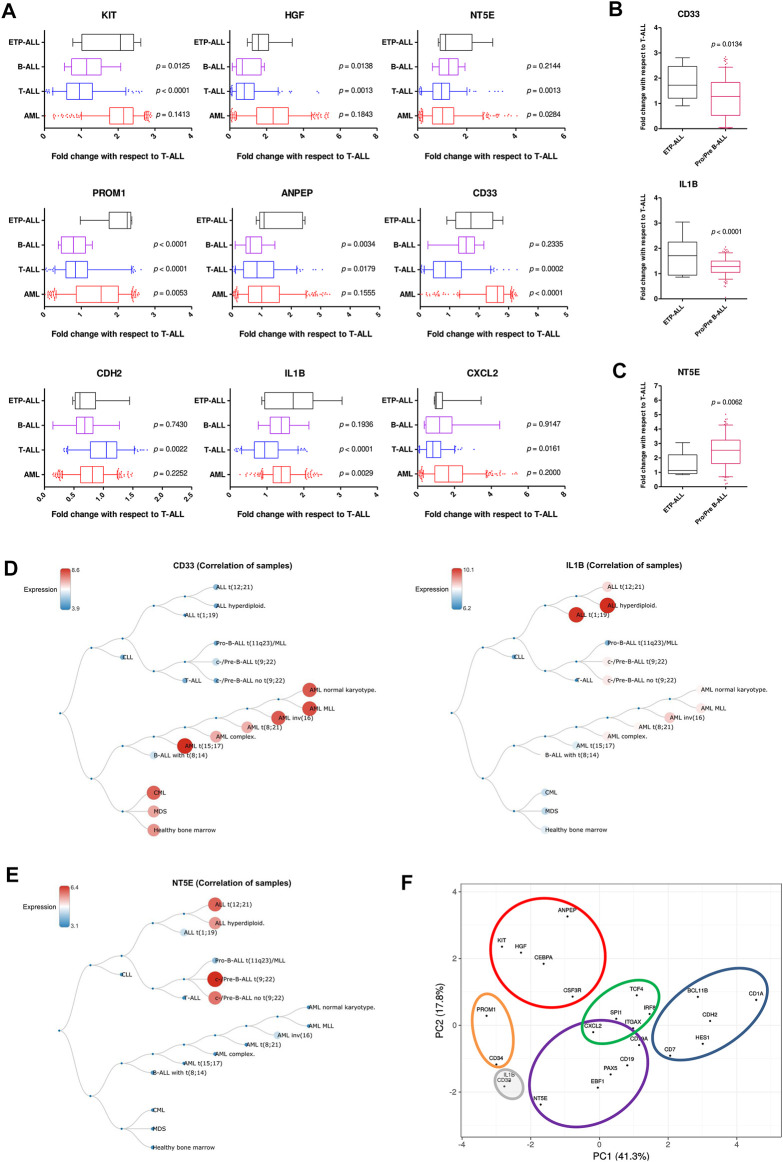
Resemblance in expression status of the proposed biomarkers in ETP-ALL and different leukemia subtypes. **(A)** Fold change in expression levels of the proposed biomarkers in ETP-ALL, B-ALL, T-ALL, and AML. **(B)** Fold change in expression levels of CD33 and IL1B in ETP-ALL and pro/pre-B-ALL. **(C)** Fold change in expression levels of NT5E in ETP-ALL and pro/pre-B-ALL. For **(A–C)**, Expression levels of the genes in ETP-ALL were derived from GSE28703 and normalized with respect to their mean expression values in non-ETP-ALL, while expression levels of the genes in pro/pre-B-ALL, B-ALL, T-ALL, and AML were derived from GSE13159 and normalized with respect to their mean expression values in T-ALL. **(D)** Correlation dendrogram of CD33 and IL1B expression levels across different leukemia subtypes from GSE13159. Constructed on BloodSpot. **(E)** Correlation dendrogram of NT5E expression levels across different leukemia subtypes from GSE13159. Constructed on BloodSpot. **(F)** PCA plot of the proposed biomarkers and standard lineage markers (from Figure 6A) with lineage demarcation boundaries (orange: stemness, purple: B-lineage, blue: T-lineage, green: dendritic cell, red: myelo-monocytic, grey: unclear lineage skew), drawn on the basis of the consolidated outcomes. Two-tailed unpaired Student’s t-test was used to calculate the statistical significance, wherever applicable.

Understanding the developmental predisposition of the leukemic blasts from ETP-ALL patients might potentiate the use of existing treatment modalities targeting lineage-restricted, mature acute leukemia against ETP-ALL. Considerable consistency was observed in terms of lineage-skewed expression of myeloid-biased *KIT*, *HGF*, *ANPEP*; B-lineage-biased *NT5E*; T-lineage-biased *CDH2* and myeloid plus B-lineage-biased *CXCL2* (Figure 7A) in ETP-ALL (from GSE28703; normalized to non-ETP-ALL) and in B-ALL, T-ALL and AML (from GSE13159; normalized to T-ALL). For example, the extent of expression of the myeloid-skewed marker *KIT* in ETP-ALL was comparable to that in AML, and was significantly higher than those in non-myeloid leukemia such as B-ALL and T-ALL, further validating the correlation between its expression and myeloid lineage bias. Despite no correlation with any B-lineage marker, levels of both *CD33* and *IL1B* in ETP-ALL were comparable with those in B-ALL. However, their levels were significantly higher in ETP-ALL than in progenitor/precursor-B-ALLs (Figure 7B). Albeit *NT5E*, their close neighbour on the PCA plane, exhibited expressional similitude between ETP-ALL and B-ALL, its levels in pro/pre-B-ALL groups were higher than those in ETP-ALL (Figure 7C), highlighting possible differences in lineage orientation of these biomarkers. Among all leukemia subtypes, *CD33* exhibited a robust enrichment in AML and CML (chronic myelogenous leukemia), while *IL1B* correlated best with pediatric ALL and considerably with AML (Figure 7D). *NT5E*, on the other hand, was not enriched in any malignancy of myeloid origin (Figure 7E). Agglomerating all these information, distinct boundaries were drawn on the 2D PCA plane, demarcating the developmental inclinations of the component genes (Figure 7F). *CDH2* and *NT5E* belonged to the T-lineage (blue) and B-lineage (purple) clusters, respectively; while *PROM1* existed in the stemness (orange) cluster. Unlike *KIT*, *HGF,* and *ANPEP* belonging exclusively to the myelo-monocytic (red) cluster, *CXCL2* was present in the overlapping zone between B-lineage and dendritic cell-lineage (green) clusters. *CD33* and *IL1B* were assigned to a separate cluster of uncertainty (grey) owing to their promiscuity in terms of lympho-myeloid specification. Of note, this grey cluster was located right between the B-lineage and stemness clusters, suggestive of their inclination towards a state in between stem cell-like pluripotency and lineage induction.

### Hub Gene Expression Might Predict the Net Lineage Bias of the ETP-ALL Leukemic Blast Populations on a Personalized Level

**FIGURE 8:**
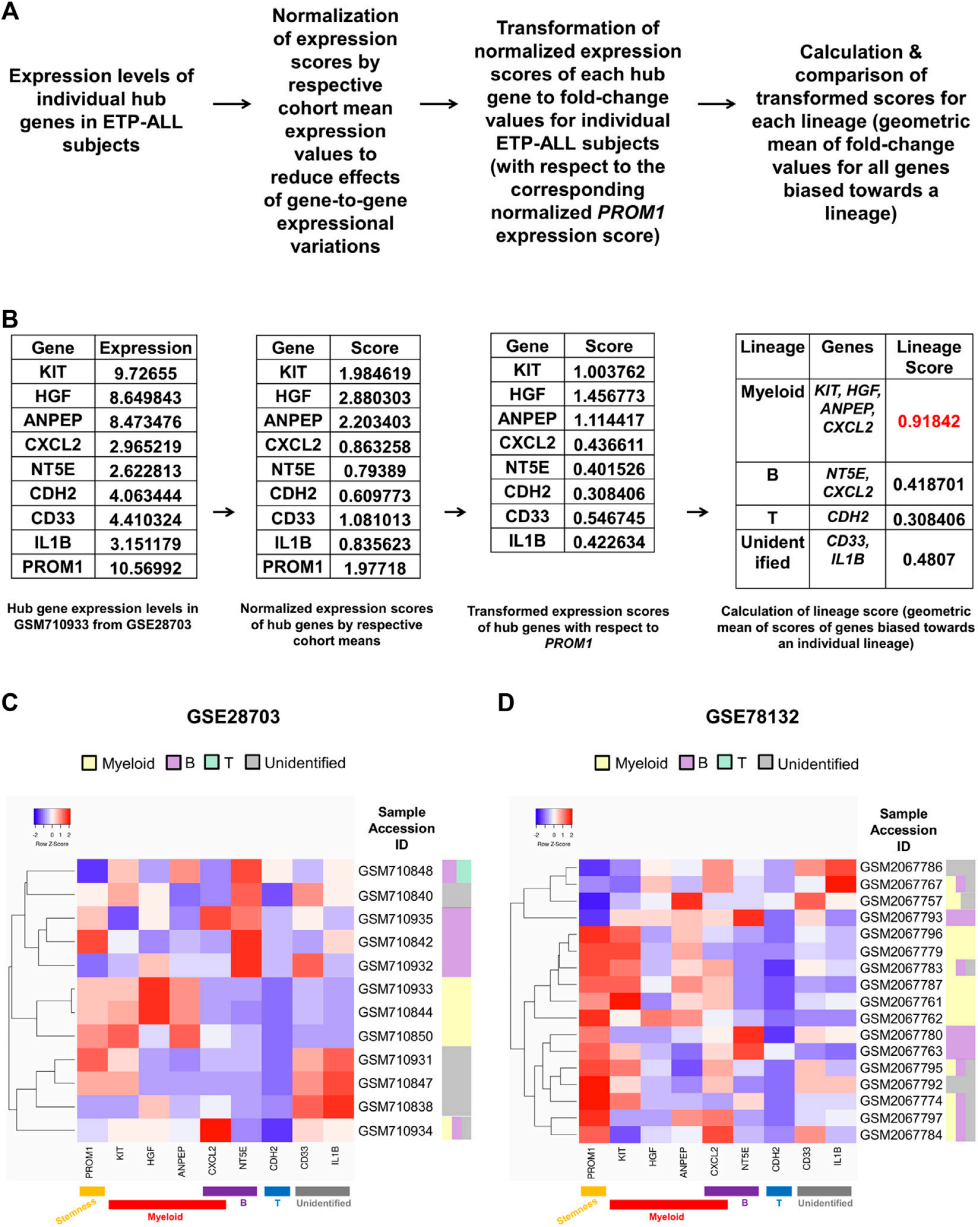
Formulation of a personalized score signifying the net lineage bias of the ETP-ALL blasts. **(A)** Mathematical derivation of the lineage score from the expression values of hub genes from individual patient samples. **(B)** Calculation of lineage score for the GSM710933 sample from the GSE28703 dataset. The lineage with the highest lineage score (labeled in red) was assigned to the sample. **(C,D)** Predictive lineage assignments for each sample from GSE28703 **(C)** and GSE78132 **(D)** based on the lineage score calculation.

Owing to the substantial inclination of the hub genes towards different hematopoietic lineages, their collective expression levels might be considered hallmark of the net developmental bias of the entire leukemic blast population in individual ETP-ALL subjects. However, manual predictions in such cases might be erroneous and highly biased. Therefore, the expression scores of all these potential ETP-ALL biomarkers were put into a single quantitative frame (Figure 8A), which would enable automated prediction of the net lineage bias of the leukemic blasts on a personalized basis. As ETP-ALL involves leukemic conversion of multipotent stem-like hematopoietic cells, expression level of the stemness marker *PROM1* was selected as reference standard for transformation of the expression levels of individual hub genes into comparable scores. Furthermore, transformed scores for different lineages for individual patients were calculated by considering the geometric means of the personalized transformed score(s) of all the markers inclined towards individual lineages. For example, normalization and transformation of the hub gene expression levels in the GSM710933 sample from the GSE28703 ETP-ALL cohort revealed a strong myeloid bias within the leukemic blasts (Figure 8B). Continuing this trend, the proposed framework indicated the net lineage inclination in every single ETP-ALL sample from the GSE28703 (Figure 8C) and GSE78132 (Figure 8D) datasets. While many samples displayed a robust skew towards the myeloid lineage, a notable proportion of the ETP-ALL cases showed considerable B-lineage bias. Interestingly, many samples exhibited a mixed skew towards myeloid and B-lineages as well as the unidentified process associated with *CD33* and *IL1B*, indicating the existence of individual ETP-ALL patients with a spectrum of multiple lineage inclinations.

### B-Lineage-Oriented Interactome in ETP-ALL Exhibits Exclusive Compartmentalization Within the Hub Gene Physical Interaction Network

**FIGURE 9:**
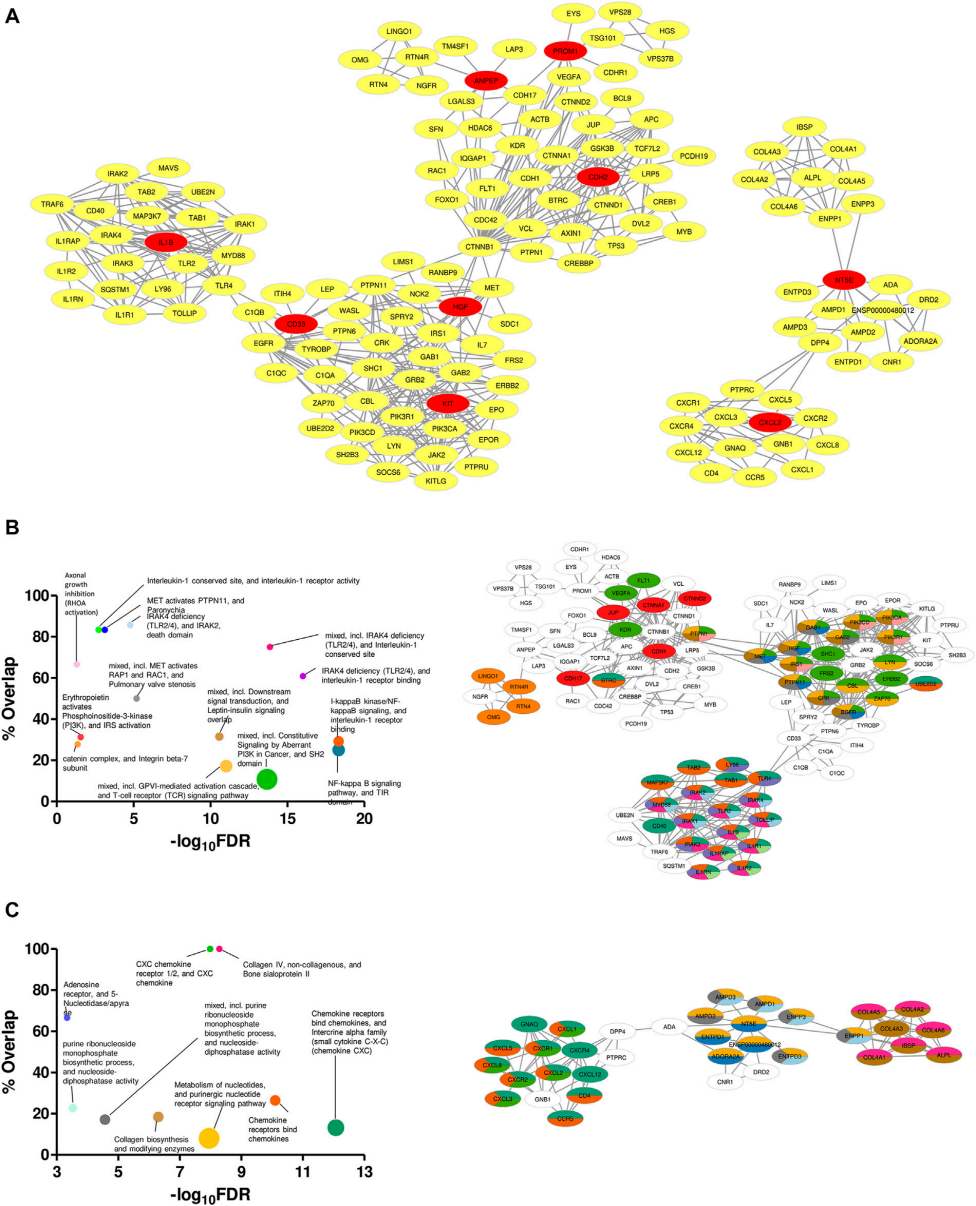
Unique clustering pattern within the interactome of the proposed biomarkers. **(A)** Merged physical interactome of the proposed biomarkers. The biomarker genes are denoted by red nodes, while the primary and secondary interactors are denoted by yellow nodes. **(B,C)** Bubble plots (**B**,**C**: left panel) depicting over-represented pathways in the left **(B)** and right **(C)** cluster, respectively. Individual pathways were mapped onto the participant genes (**B**,**C**: right panel). Sizes of the bubbles on the bubble plots are directly proportional to the gene set sizes. Colours on the pathway-mapped networks denote individual pathways, as colour-coded on the corresponding bubble plots.

Specificity in expression of the proposed markers in ETP-ALL of different lineage inclinations not only supported their presumed lineage bias, but also raised possibilities for these markers and the nexus of their associated proteins to be used directly as druggable targets against cases of ETP-ALL with specific developmental skew(s). Therefore, mining the symbiosis among the total physical interactome of these genes was of utmost importance, as any mode of therapeutic interventions against them is expected to perturb the functioning of all these interactors together. Although enrichment analyses based on the physical interaction networks of the individual genes unveiled the downstream functions performed by their individual interactomes (Supplementary Figure S8), this was not enough to recognize the relative overlap among them. Interestingly, the converged physical interactome comprising the proposed biomarkers along with their primary and secondary interactors (Figure 9A) exhibited two distinct clusters. The larger cluster consisted of hub genes with different lineage propensities, while the smaller cluster contained only two of the proposed biomarkers: *NT5E* and *CXCL2*, the only genes having strong B-lineage inclination. The larger cluster was enriched in an assorted list of pathways (Figure 9B, left panel), where individual pathways were mapped in an overlapping fashion, onto interactors of different hub genes (Figure 9B, right panel). The smaller cluster over-represented mostly three events: purine nucleotide signaling, collagen metabolism and CXCR1/2 signaling (Figure 9C, left panel). The pathway mappings were well-separated among the interactors of the two hub genes (Figure 9C, right panel), indicating their non-redundant actions towards B-lineage priming.

## Discussion

Analysis of the cellular transcriptome is widely used to generate holistic ideas about disease pathogenesis and pinpoint critical markers for their diagnosis, prognosis and therapeutic targeting (Casamassimi et al., 2017). In this study, analyses of the ETP-ALL transcriptome unveiled a pool of genes whose expression levels might act as binary sorters between ETP-ALL and non-ETP-ALL within an ALL cohort and simultaneously predict the specification bias of the leukemic blasts from individual ETP-ALL patients. The study eventually proposes a novel transcriptional phenotype of ETP-ALL as: *KIT* ^+^ *HGF* ^+^ *ANPEP* ^+^ *NT5E* ^+^ *CDH2* ^−^ *PROM1* ^+^ *CXCL2* ^+^ *CD33* ^+^ *IL1B* ^+^, which might be used in conjunction with the conventional biomarkers (Coustan-Smith et al., 2009). The race and ethnicity-dependent variations in global diversity of ETP-ALL (Hunger and Mullighan, 2015) must be taken into account while considering an optimum cut-off for the proposed diagnostic markers in distinguishing ETP-ALL from other ALLs. One strategy to avoid this issue might be to normalize the gene expression scores with respect to the corresponding population mean (as done in the present work) before fixing a diagnostic cut-off, thereby reasonably nullifying the race and ethnicity-associated variations. Of note, ANPEP and CD33 are already enlisted as immunophenotypic ETP-ALL markers (Jain et al., 2016). KIT, NT5E, PROM1, and CDH2 are cell surface proteins, which makes them potentially eligible for being used in immunophenotypic identification of ETP-ALL. Besides, HGF, IL1B, and CXCL2 are potential soluble markers of ETP-ALL due to their secreted nature. Assuming a linear relationship between their observed transcript levels and expected protein levels in these contexts, all these markers might therefore be added to the existing panel of ETP-ALL biomarkers for multiparametric flow cytometry-based immunophenotypic analyses. Use of this updated panel would not only increase the specificity in diagnosis of ETP-ALL, but would also facilitate early detection of the inherent lineage bias of the ETP-ALL blasts on a personalized basis, effecting prompter and more specific actions by the clinicians.

Despite recent developments in single-cell RNA sequencing explicating the intra-population heterogeneity in ETP-ALL (Anand et al., 2021) as well as ETPs (Zhou et al., 2019; Lavaert et al., 2020), this study did not investigate the ETP-ALL transcriptome on a single-cell resolution. An individual ETP-ALL patient might (and often do) harbour a heterogeneous community of leukemic blasts due to their underlying multipotency (Kumar et al., 2019). Since therapeutic modules targeted against ETP-ALL would act against the entire community of neoplastic lymphoblasts, this study focussed onto the single subject-level transcriptome of ETP-ALL to establish a platform for precision medicine against the dominant leukemic blast subpopulations in individuals. The proposed ETP-ALL markers identified by this study, showing diverse propensities towards different developmental outcomes, might act as hallmarks of lineage priming in ETP-ALL blasts. Consequently, their expression levels in an individual patient might evoke an idea of the net developmental inclination of the entire leukemic blast population in that patient (Figure 8). *GATA3*, one of the top 30 hub genes identified by this study (Figure 3E), has been reported to split the ETP-ALL patients into subpopulations with different potencies depending upon its expression (Fransecky et al., 2016). This study, in addition to all related studies done so far, provides with a mathematical framework for the detection of potential lineage inclinations on a personalized level. Thus, more such subpopulations within the ETP-ALL patient cohorts might be identified on the basis of such multi-parametric analyses, which might improve the clinical outcome of ETP-ALL to a significant extent by opening new avenues for large-scale drug repurposing.

The incompletely differentiated ETP-ALL blasts are not true mimics of the differentiated leukemic blasts from B-ALL or AML; resonated by their high levels of stem cell-associated gene expression (Coustan-Smith et al., 2009). Therefore it is not easy to devise lineage-targeted therapies against them. Targeting random non-T-lineage markers of mature hematopoietic cells such as CD14 or CD19 in case of ETP-ALL might not be fruitful because of their incompletely differentiated status, where the blasts might not abundantly express those particular lineage markers. Contrarily, expression of the biomarkers proposed in this study varies considerably across ETP-ALL subjects. Distances along the first principal component on the PCA plane (Figure 7F) signified greater disparity between the expression patterns of *CD34* and the mature non-T-lineage markers (such as *CSF3R*, *CD19*), compared to those between *CD34* and the corresponding lineage markers proposed in this study (such as *KIT*, *HGF*, *NT5E*). This indicates the ETP-ALL blasts to exist in an intermediate state between absolute stemness and complete lineage fixation, where the expression levels of the proposed markers might act as better indicators of specification than the levels of the mature-stage lineage markers. Close inspection of the physical interactome of the proposed markers, hence, might be profitable in defining the cellular processes associated with the lineage-priming checkpoints within ETP-ALL, which might be ideal therapeutic targets. Since some of the markers are even cell-surface expressed, they might also be exploited for targeted delivery of therapeutics specifically to the ETP-ALL blasts. In fact, CD33-targeted delivery of IMGN779 against ETP-ALL *in vitro* has already yielded promising results (Khogeer et al., 2019), making such superficial biomarkers lucrative targets for the delivery of therapeutics.

The considerable B-lineage inclination in a sizeable fraction of ETP-ALL samples (Figures 8C,D) coupled with the isolated presence of the B-lineage-related cluster within the entire interactome associated with the hub genes (Figure 9A) highlights the necessity of acknowledging the B-lineage bias of ETP-ALL with utmost importance. Because of the partition between the B- and non-B-lineage networks, therapeutic tweaking inside the non-B interactome would most likely be insufficient against B-lineage-primed ETP-ALL blasts. Incidentally, in a limited sized cohort, relapse after >100 days of remission post-chemotherapy was observed in every single ETP-ALL patient expressing B-lineage markers on the leukemic blasts (Garg et al., 2019). Till date, no chemotherapeutic protocol has exclusively targeted the B-lineage-associated pathways like adenosine receptor signaling in ETP-ALL. This under-appreciation of B-potency in selective ETP-ALL cases might effectuate the prognostic worsening as well as high relapse rates of B-lineage-inclined ETP-ALL over the others. The markers proposed in this study, thus, might facilitate the detection of specific cases of ETP-ALL with significant B-lineage predisposition along with simultaneous targeting of the primary pathways (such as adenosine signaling) encompassing the B-lineage markers.

Multiple studies have uncovered the molecular phenomena behind the lineage plasticity of ETPs, and have designated certain genes as barcodes of particular specification programmes in these multipotent cells (Chiara et al., 2021; Hosokawa and Rothenberg, 2021). However, to the best of our knowledge, this study is the first of its kind to delineate the developmental pliability of the neoplastic forms of these multipotent cells, bringing forth new opportunities to subclassify multipotent ETP-ALL samples based on their lineage bias. This strongly recommends the invention of novel therapeutic approaches as well as opens up possible prospects of repurposing existing B-ALL or AML-directed therapies on a personalized mode. In parallel, the current study suggests the presence of ETP-ALL samples with mixed lineage propensities or unidentified developmental bias, calling for further basic as well as translational research in order to formulate appropriate clinical strategies in these aspects. On top of all, such observations merit many more high-throughput studies with ETP-ALL blast samples from patients of a wide range of socio-biological factors (such as age, ethnicity etc) which affect the hematopoietic quality-control checkpoints, to gain further insights into the deeper mechanisms of leukemogenesis.

## Data Availability

The public microarray gene expression datasets used in this study are available on NCBI GEO DataSets (URL: https://www.ncbi.nlm.nih.gov/gds) against the respective accession numbers: GSE28703, GSE78132, and GSE13159. Processed data in any form, not reported in the figures/Supplementary Material, will be available from the corresponding authors upon reasonable request.
