# Supplementary material for: In Silico Integration of Transcriptome and Interactome Predicts an ETP-ALL-Specific Transcriptional Footprint that Decodes its Developmental Propensity
Source: Front Cell Dev Biol. 2022 May 13;10:899752. doi: 10.3389/fcell.2022.899752 (PMC9138408; doi:10.3389/fcell.2022.899752)
Supplement: Supplementary file 5 [file DataSheet1.PDF]

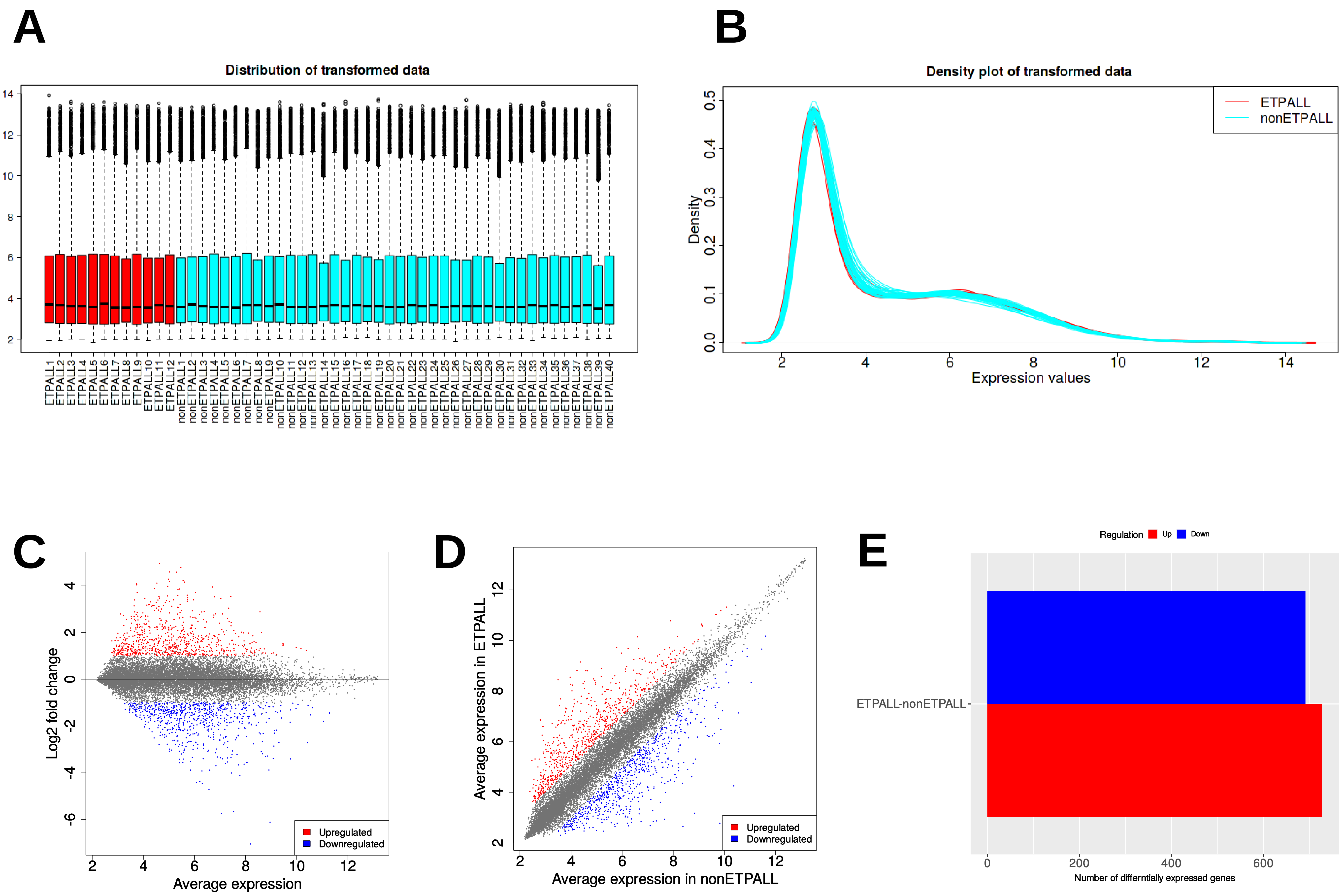

Supplementary Figure S1. Quality assurance of processed transcriptome data from GSE28703 and differential gene expression analysis. A. Box plot of expression values from GSE28703. B. Density plot of expression values from GSE28703. C. MA plot of differential gene expression in GSE28703. D. Scatter plot of average gene expression across groups in GSE28703. E. Number of differentially expressed genes in GSE28703.

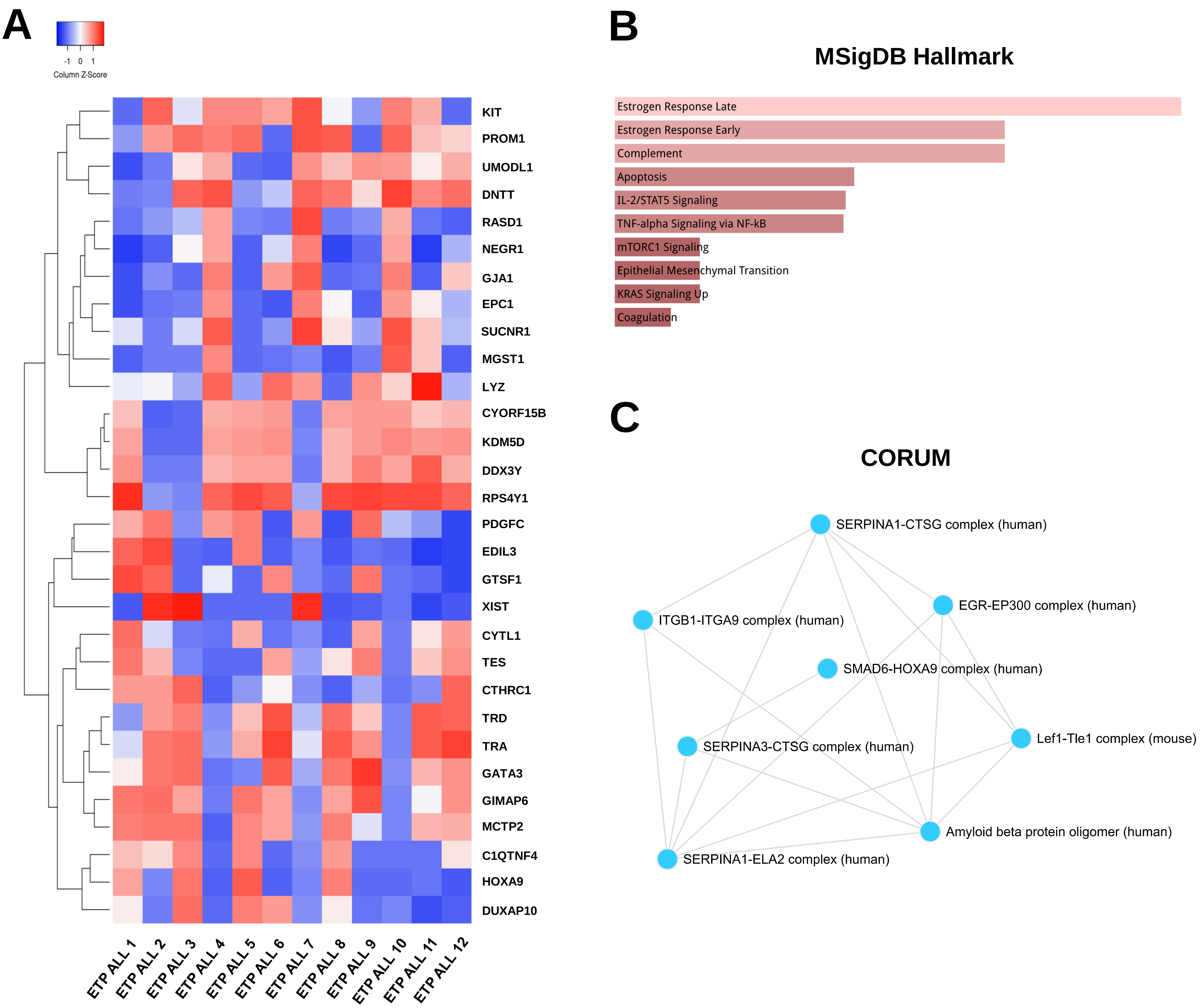

Supplementary Figure S2. Variably expressed genes in ETP-ALL and their functional implications. A. Top 30 variably expressed genes across ETP-ALL samples in GSE28703. B. Top enriched MSigDB hallmark pathways in variably expressed genes across ETP-ALL. C. Top enriched protein complexes (from CORUM) associated with variably expressed genes across ETP-ALL.

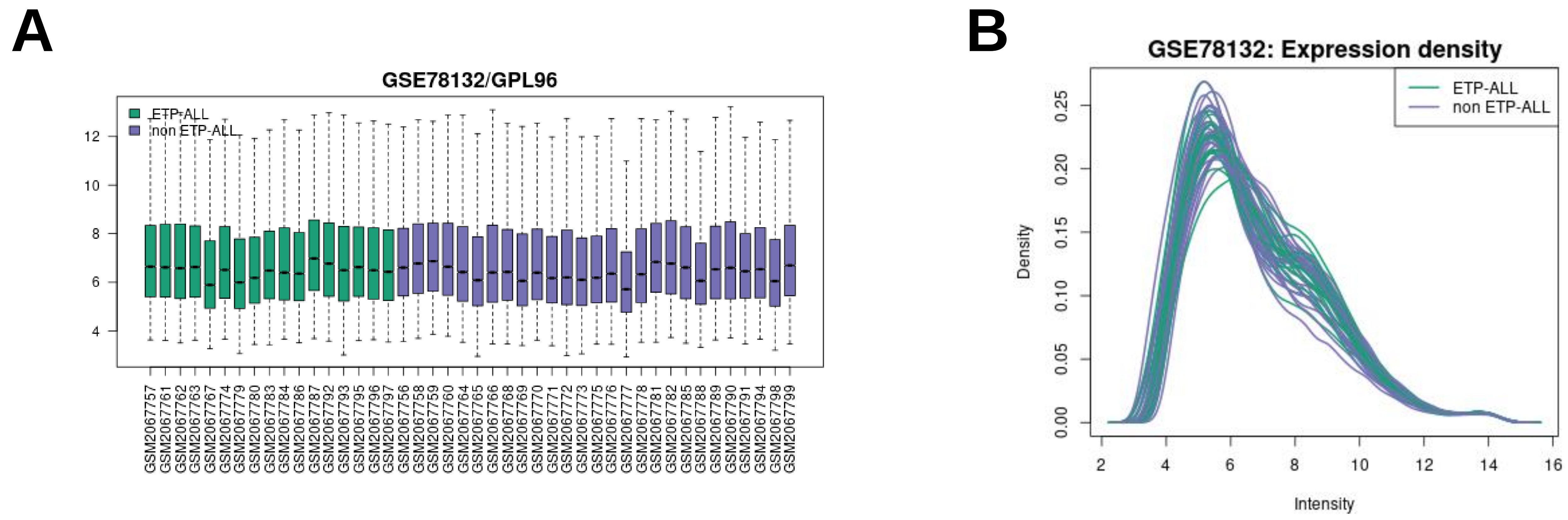

Supplementary Figure S3. Quality assurance of transcriptome data from GSE78132. A. Box plot of expression values from GSE78132. B. Density plot of expression values from GSE78132.

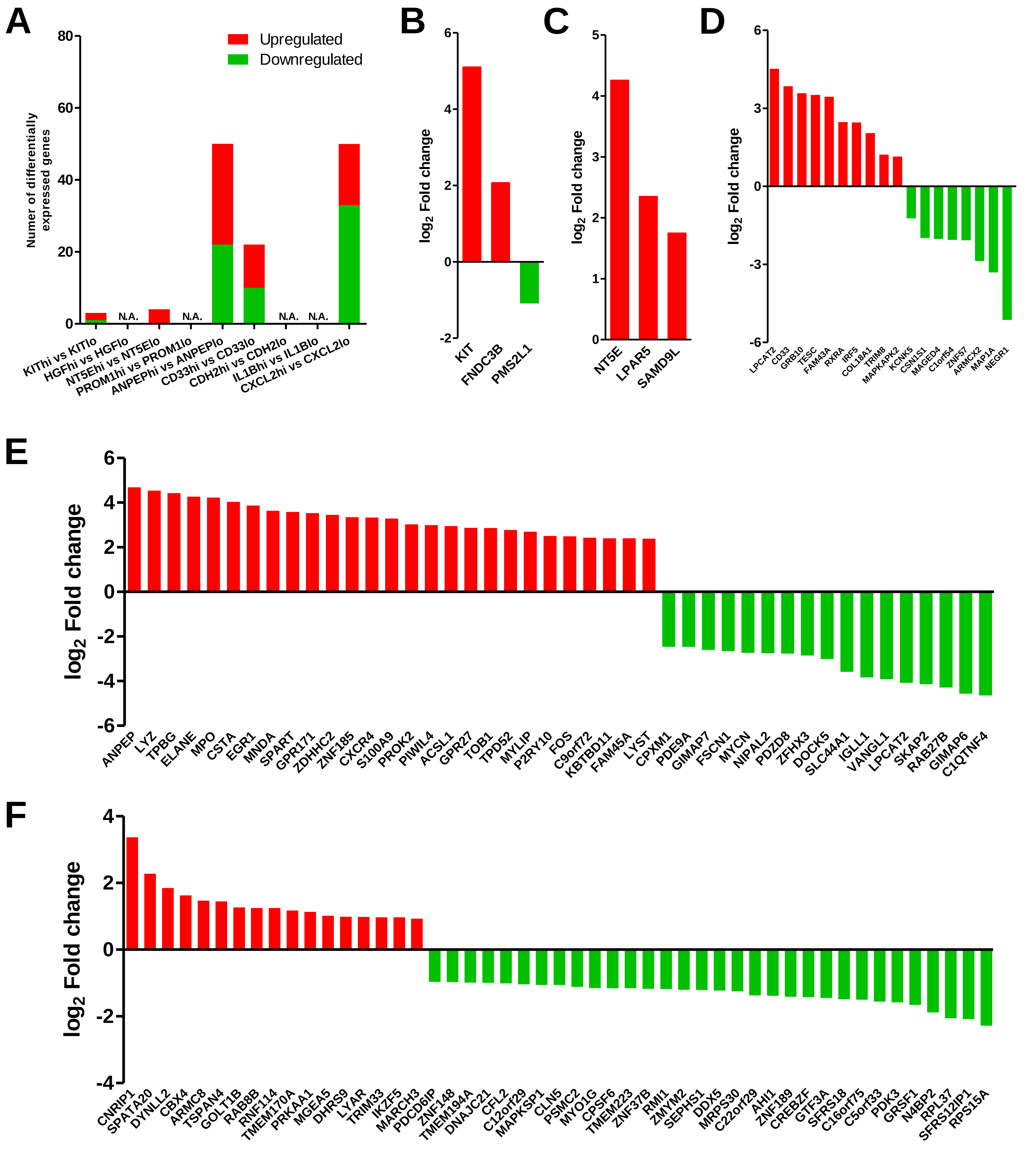

Supplementary Figure S4. Differential gene expression between ETP-ALL subpopulations with variable hub gene expression in GSE28703. A. Number of differentially expressed genes between each pair of ETP-ALL subpopulations based on different levels of hub gene expression. B-F. $\log_2$ Fold change in expression of differentially expressed genes in KIThi vs KITlo (B), NT5Ehi vs NT5Elo (C), CD33hi vs CD33lo (D), ANPEPhi vs ANPEPlo (E) and CXCL2hi vs CXCL2lo (F) comparisons.

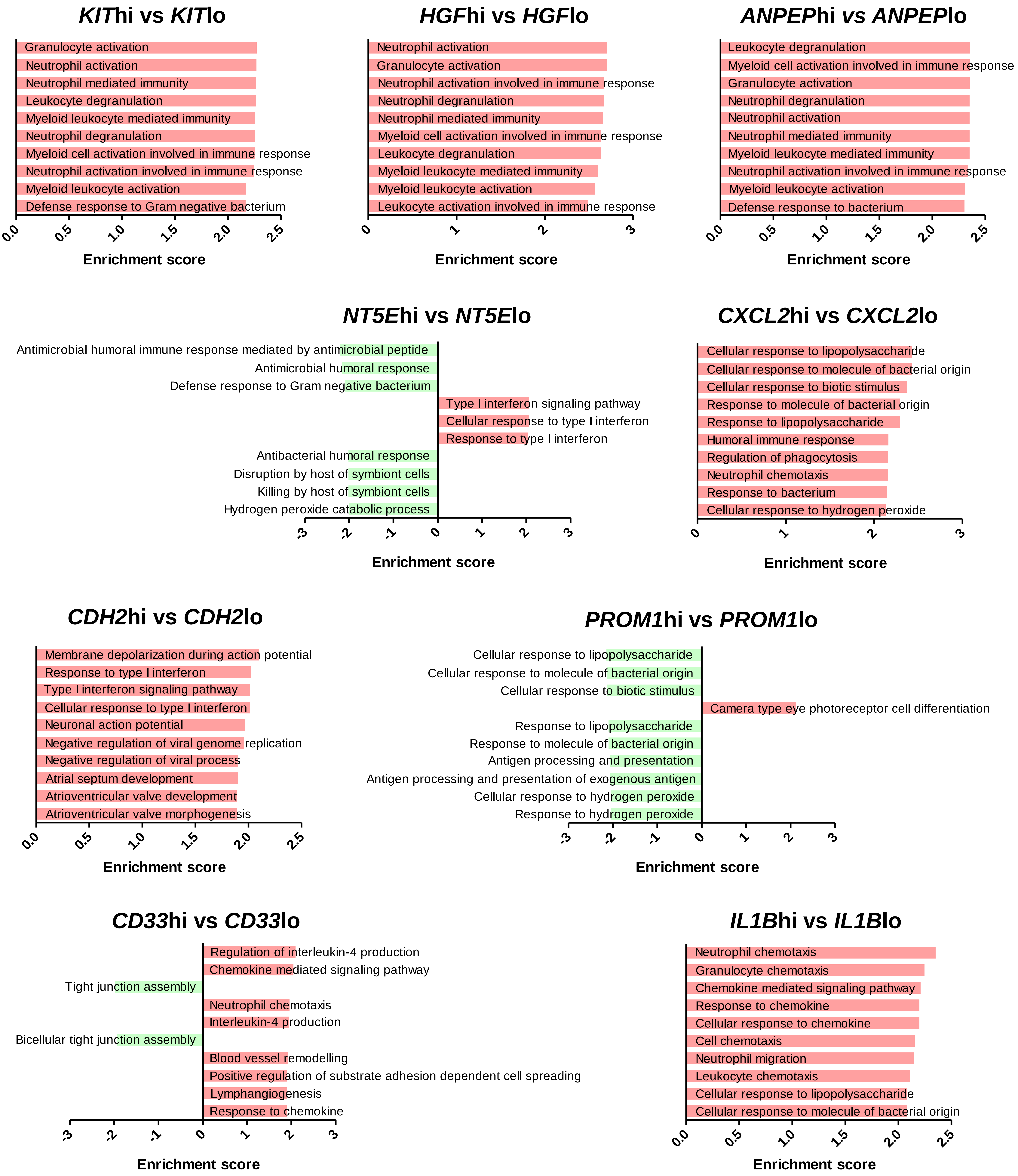

Supplementary Figure S5. Top altered pathways altered between ETP-ALL subpopulations with variable hub gene expression in GSE28703. ‘GO biological process’ database was used as reference.

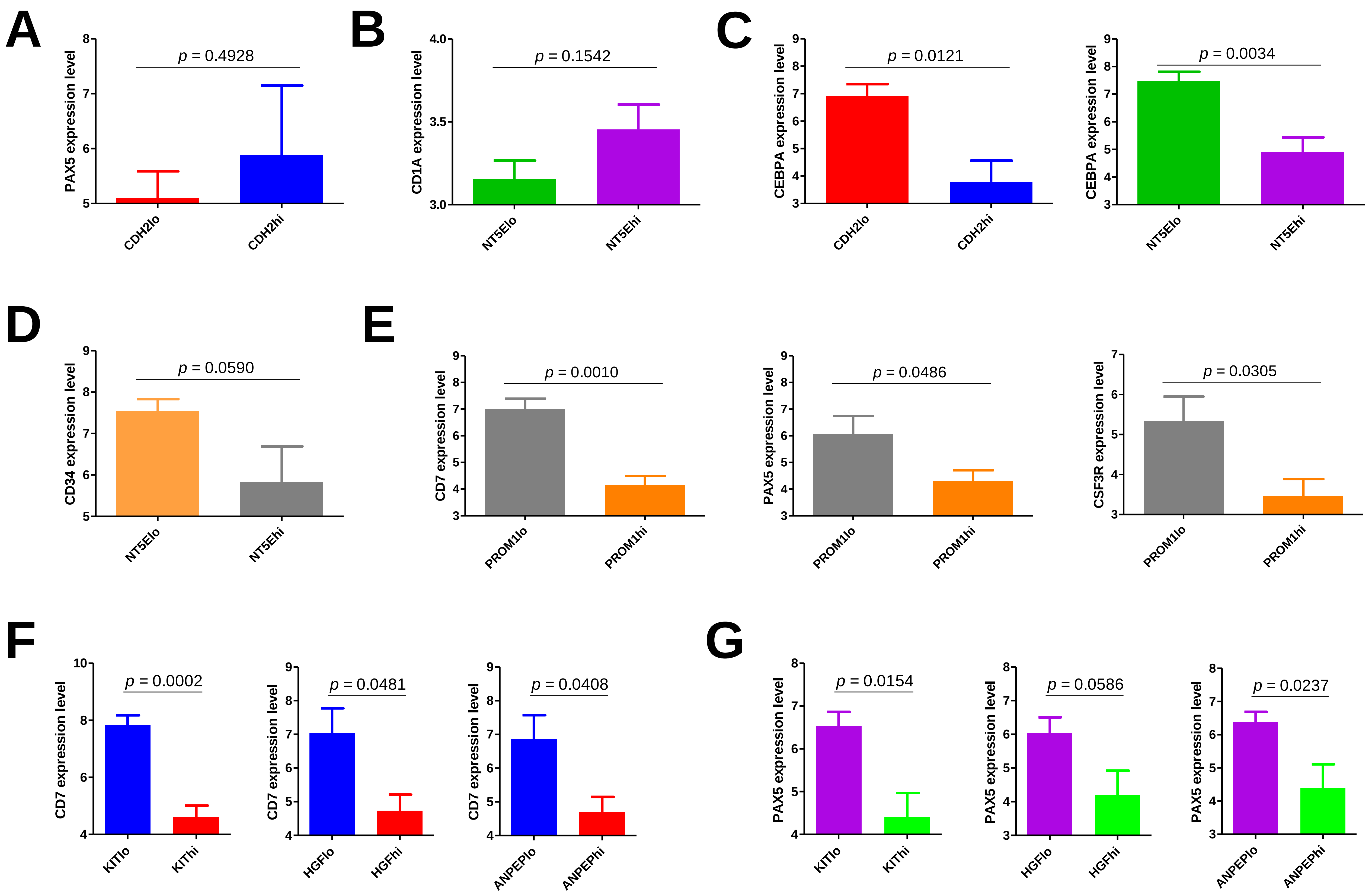

Supplementary Figure S6. Expression level of distant lineage markers with different levels of hub genes in GSE28703. A. Expression of PAX5 along with different levels of CDH2. B. Expression of CD7 along with different levels of NT5E. C. Expression of CEBPA along with different levels of CDH2 and NT5E. D. Expression of CD34 along with different levels of NT5E. E. Expression of CD7, PAX5 and CSF3R along with different levels of PROM1. F. Expression of CD7 along with different levels of KIT, HGF and ANPEP. G. Expression of PAX5 along with different levels of KIT, HGF and ANPEP. Two-tailed unpaired Student's t-test was performed between each pair of data.

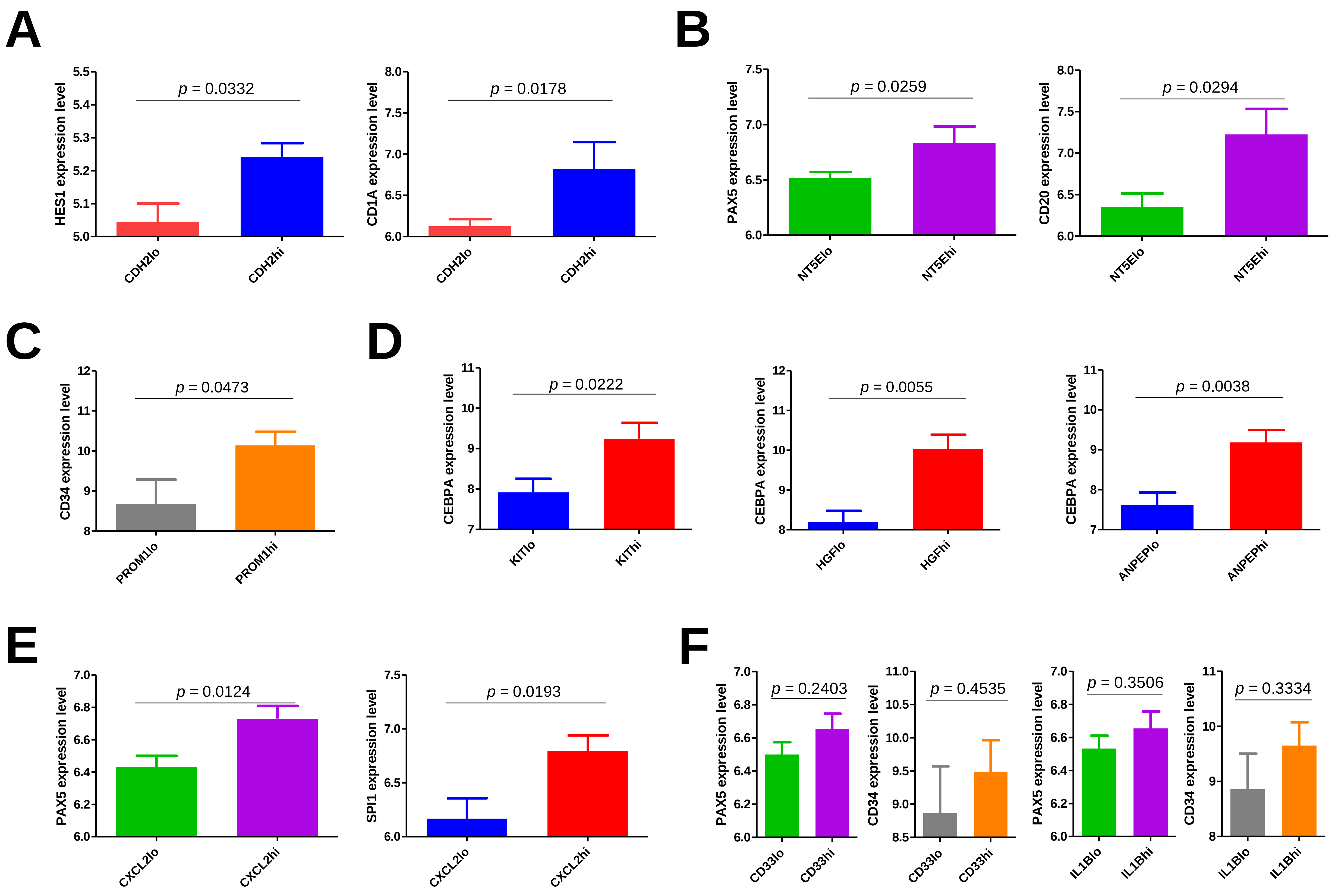

Supplementary Figure S7. Validation of lineage propensities of proposed ETP-ALL markers in GSE78132. A. Expression of HES1 and CD1A along with different levels of CDH2. B. Expression of PAX5 and CD20 along with different levels of NT5E. C. Expression of CD34 along with different levels of PROM1. D. Expression of CEBPA along with different levels of KIT, HGF and ANPEP. E. Expression of PAX5 and SPI1 along with different levels of CXCL2. F. Expression of PAX5 and CD34 along with different levels of CD33 and IL1B. Two-tailed unpaired Student's t-test was performed between each pair of data.

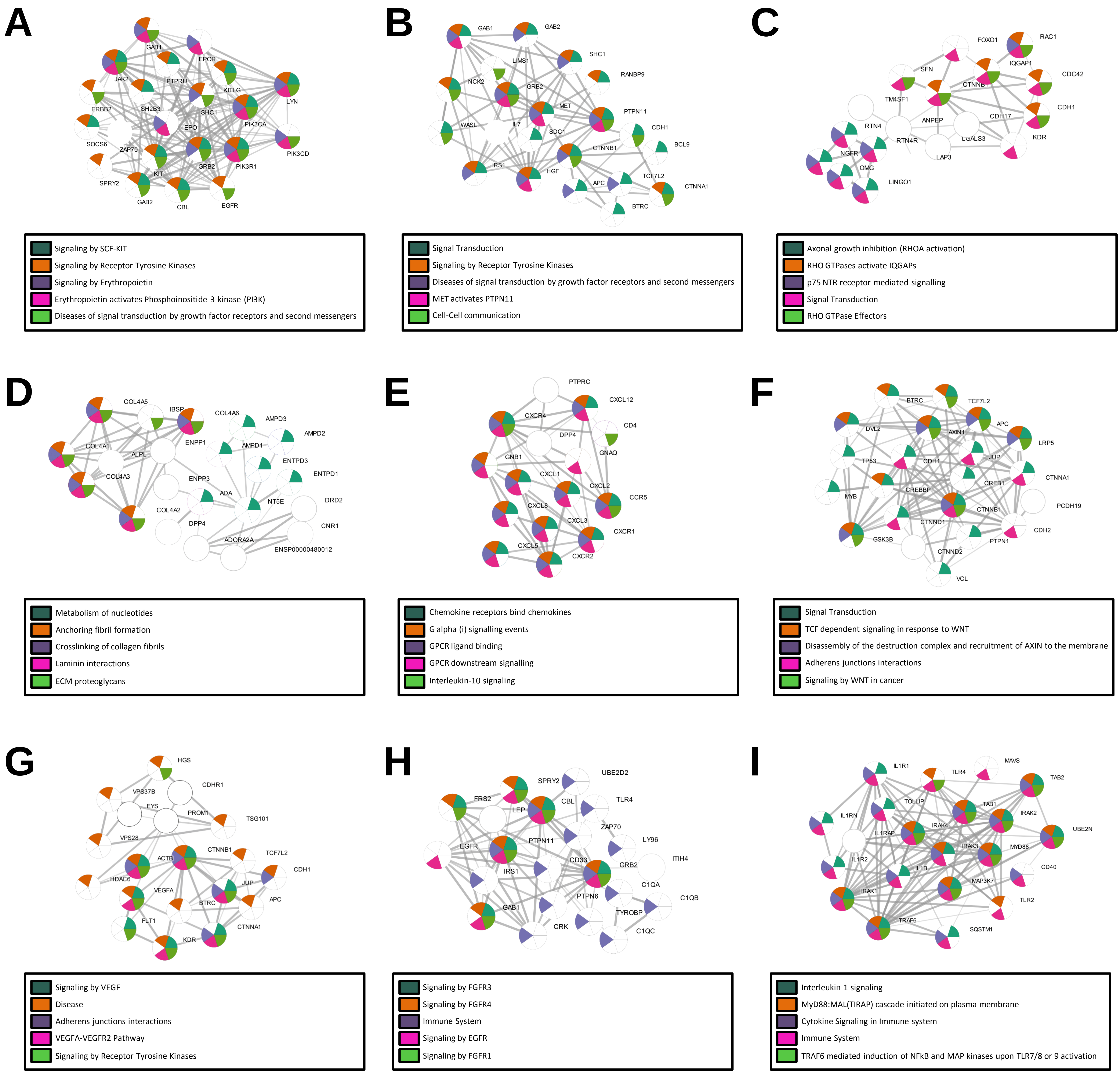

Supplementary Figure S8. Top REACTOME pathways enriched in the physical interactome of individual biomarker candidates (A. KIT B. HGF C. ANPEP D. NT5E E. CXCL2 F. CDH2 G. PROM1 H. CD33 I. IL1B). Each circle represents a protein, while coloured segments inside the circles represent association of that protein with the corresponding pathway. Colourless circles represent proteins without direct involvement in the top 5 pathways.
